# Hybrid natural language processing tool for semantic annotation of medical texts in Spanish

**DOI:** 10.1186/s12859-024-05949-6

**Published:** 2025-01-08

**Authors:** Leonardo Campillos-Llanos, Ana Valverde-Mateos, Adrián Capllonch-Carrión

**Affiliations:** 1 ILLA - CSIC (Spanish National Research Council), C/Albasanz 26-28, 28037 Madrid, Spain; 2 Medical Terminology Unit, Spanish Royal Academy of Medicine, C/Arrieta 12, 28013 Madrid, Spain; 3 Centro de Salud Retiro, Hospital Universitario Gregorio Marañon, C/Lope de Rueda, 43, 28009 Madrid, Spain

**Keywords:** Medical natural language processing, Medical text mining tool, Named entity recognition, Deep learning in healthcare, Clinical trials, Spanish medical NLP

## Abstract

**Background:**

Natural language processing (NLP) enables the extraction of information embedded within unstructured texts, such as clinical case reports and trial eligibility criteria. By identifying relevant medical concepts, NLP facilitates the generation of structured and actionable data, supporting complex tasks like cohort identification and the analysis of clinical records. To accomplish those tasks, we introduce a deep learning-based and lexicon-based named entity recognition (NER) tool for texts in Spanish. It performs medical NER and normalization, medication information extraction and detection of temporal entities, negation and speculation, and temporality or experiencer attributes (Age, Contraindicated, Negated, Speculated, Hypothetical, Future, Family_member, Patient and Other). We built the tool with a dedicated lexicon and rules adapted from NegEx and HeidelTime. Using these resources, we annotated a corpus of 1200 texts, with high inter-annotator agreement (average F1 = 0.841% ± 0.045 for entities, and average F1 = 0.881% ± 0.032 for attributes). We used this corpus to train Transformer-based models (RoBERTa-based models, mBERT and mDeBERTa). We integrated them with the dictionary-based system in a hybrid tool, and distribute the models via the Hugging Face hub. For an internal validation, we used a held-out test set and conducted an error analysis. For an external validation, eight medical professionals evaluated the system by revising the annotation of 200 new texts not used in development.

**Results:**

In the internal validation, the models yielded F1 values up to 0.915. In the external validation with 100 clinical trials, the tool achieved an average F1 score of 0.858 (± 0.032); and in 100 anonymized clinical cases, it achieved an average F1 score of 0.910 (± 0.019).

**Conclusions:**

The tool is available at https://claramed.csic.es/medspaner . We also release the code (https://github.com/lcampillos/medspaner) and the annotated corpus to train the models.

## Introduction

The substantial volume of medical data contained within electronic health records (EHRs), articles or clinical trials represents a potential source of evidence and knowledge discovery [1, 2]. However, information is predominantly stored in a unstructured format, which poses challenges for effective extraction and analysis. The application of natural language processing (NLP) techniques has facilitated more efficient text mining in the medical domain [3].

Comprehensive NLP tools can extract relevant information and alleviate the manual curation of data by healthcare professionals, which is time-consuming and error-prone [4]. Such type of system can automate cohort definition tasks for clinical trials by extracting medical concepts from eligibility criteria [5], and can enhance the discovery of disease-drug pairs for drug-repurposing [6, 7]. Likewise, it might be used for information extraction from clinical cases, with the goal of enhancing data analysis in large volumes of data [8] and identifying phenotype variance [9]—a step towards precision medicine [10]. Potential applications range from automatically extracting co-occurrence of drug-disease pairs in real data [11, 12], detecting comorbidities, disease recurrence and risk factors (e.g., in dementia [13], cardiovascular [14, 15] or mental health [13, 16, 17])—or EHR-based pharmacovigilance [18]. The reuse of clinical data can also be employed to identify potential participants who meet the eligibility criteria of clinical trials [19]. In these tasks, the detection of negation and speculation is essential for accurately excluding pathological states and comorbidities that are not associated with specific conditions [20]. Additionally, the processing of temporal information and the history of medical conditions is critical for predicting clinical events and managing chronic diseases [21].

A challenge in implementing this type of NLP system is the considerable effort required to tailor it specifically to each use case [22]. Ideally, an open-source medical NLP tool should be flexible enough to allow other teams to customize it for their particular needs. Additionally, such a system should be capable of processing a wide range of information, encompassing disorders, treatments or age-groups, negated and speculated events, drug contraindications and history of conditions or procedures. Although there has been a rise in the number of teams conducting research in Spanish medical natural language processing (NLP), integrated systems remain limited. A notable exception is the framework developed by [23], which facilitates the expansion of medical terminologies, enabling the reuse of information available in EHRs for advanced data analysis.

In this context, we introduce MEDSPANER, a Medical Semantic Python-Assisted Named Entity Recognizer for the Spanish language. The tool performs medical named entity recognition (NER), medication information extraction, temporal entity annotation and detection of negation and speculation. The system also normalizes concepts according to Concept Unique Identifiers (CUIs) from the Unified Medical Language System (herein, UMLS) [24] or SNOMED CT codes [25], using a dedicated lexicon  [26].

**Fig. 1:**
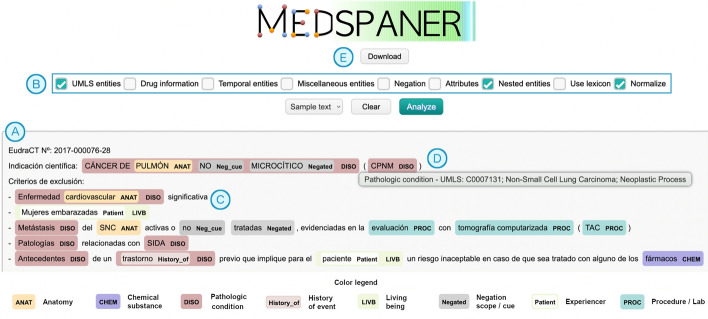
Graphical User Interface. A text is copied on the text box (**A**) and several annotation options can be selected (**B**). The annotated entities are shown with different colors (**C**). Hovering over an entity shows information about its type and normalization (e.g. UMLS CUIs), if available (**D**). Results can be downloaded in BRAT format (**E**)

We developed the tool to automate the processing and analysis of clinical trials by medical professionals. While it was initially designed for this specific text genre, we are releasing it with the intention that it can also be applied to other medical sub-genres or adapted for additional tasks in the future. A demo video is provided as supplementary material. Our contributions are:

- An integrated tool that can be used with a UNIX-based terminal, or with a graphical user interface (GUI; Fig. 1). Annotations can be normalized to UMLS CUIs or SNOMED CT codes. The output can be json (which enables exporting data to a database such as Mongo DB) or the ann format (which enables loading the annotated files in the BRAT tool [27]).
- A set of fine-tuned Transformer-based models already available for the above-mentioned sub-tasks.
- An error analysis of the system’s output, and a human evaluation by medical professionals, to show its strengths and limitations.
- An enriched annotation of the CT-EBM-SP corpus [28], used to develop the tool and to train the deep learning models. We also share the 200 annotated texts used in the human evaluation.

A supplementary graphical abstract summarizes the contents of this work [see Additional-File-1].

## Background

NLP libraries in the Python language—e.g., Stanza [29] or spaCy [30]—currently perform fast automatic tokenization, sentence splitting and linguistic analysis (part-of-speech, syntax or semantic tagging). In addition, advances in neural-network-based frameworks [31, 32] provide modules to integrate the output of those linguistic analyses with contextual information available in pre-trained language models [33] or contextual embeddings [34]. In summary, the current NLP ecosystem enables the construction of complex pipelines for the processing of medical language. Some examples of Python libraries are those developed to lookup the UMLS Metathesaurus [35], for processing EHRs or other clinical texts [36–41].

Multipurpose medical annotation tools feature medical entity annotation and classification—e.g., MedLEE [42], MetaMap [43] or MetaMap Lite [44]—and also negation and assertion detection—e.g., cTAKES [4]; or CLAMP [22]. For medical entity recognition, systems can use dictionaries [43]. These have the advantage of identifying entities in unsupervised contexts, and cope with infrequent term mentions, e.g., microbial species [45, 46]. Lexicon methods can be combined with rules and machine learning [4]. Recently, general models have been adapted or pre-trained on large scale biomedical and clinical data [47–50]. Recent experiments have tested dictionary-based approaches to feed a lexicon output into the neural model [51, 52].

With regard to temporal information [53], rules such as HeidelTime [54] or machine-learning classifiers have been applied, which yielded good results with clinical texts [55, 56]. Similar methods were used to process temporal entities and relations in clinical trials [57, 58]. For the detection of negation, uncertainty, temporality and experiencer attributes, methods have evolved from rules—e.g., NegEx [59] and ConText [60]—to supervised neural-network-models [61–65]. For the extraction of medication information, a similar transition has occurred from rule-based systems to neural-based or hybrid approaches  [66, 67].

All the same, current advances of large language models (LLMs) and GPT-based methods are been applied to parse trial eligibility criteria and match patients to EHR data [68, 69]. Nonetheless, privacy issues to keep patients’ protected information in each health institution poses an major limitation to commercial systems such as ChatGPT. In this context, Transformer-based models and open-source projects are a feasible alternative, given that they can be executed locally, and their results are competitive—sometimes with higher performance, according to some studies  [70]. Combining such type of framework and a physician-in-the-loop to check the system output [71] seems a suitable approach.

Most tools are available for the English language and few tools are aimed at eligibility criteria of clinical trials [5] (no system exists for the Spanish language, to the best of our knowledge). Numerous teams have contributed to the task of medical entity recognition in Spanish; herein, we review only the works most related to ours (Table D.9 in Appendix D gathers more details). Initiatives include detecting drug effects in social media [72], drugs in clinical cases [73], or adverse drug reactions in EHRs by applying random forests (RFs) [74]. ICD-10 coding of disorders in EHRs was explored using RFs [75] or BERT models on radiology reports [76]. Also in radiology reports, a wide range of entities such as findings or body parts were annotated [77]. Detecting disabilities in Orphanet data was tested by means of SVM classifiers or Bi-LSTM networks [78] and BERT models [79]. In clinical referrals, entities were extracted by using a Bi-LSTM-CRF framework [80]; the same method combined with rules was applied to health reports of disease outbreaks [81]. Temporal entity recognition was performed by means of Bi-LSTM-CRF architectures in clinical narratives [82] or RoBERTa models in clinical cases [83]. As far as we know, detecting medication information (e.g. dosage) in Spanish was only explored on summaries of product characteristics by employing dictionary-based methods [84]. Negation detection was performed using syntactic methods on radiology reports [85], and negation/uncertainty was detected by applying Bi-LSTM-CRF or BERT-based models on product reviews, journal articles and clinical notes [62, 64, 65].

Overall, most methods have relied on Bi-LSTM networks or BERT models in the latest years. However, no comprehensive system exists for fast and actionable processing of that type of relevant information, and most works have focused on specific semantic aspects. No open-source tool has been developed that integrates annotation models for medical entities, negation and uncertainty, temporal information, and experiencer within a unified framework for Spanish medical texts. In addition, medical attributes of event temporality (e.g. History_of) or experiencer (e.g. Patient or Family_member) have not been considered in other NLP projects for the clinical literature in Spanish.

This work contributes with an integrated tool for medical language processing of texts in Spanish language. It extracts UMLS entities, medication-related data, temporal information, negation or speculation, experiencer or event temporality attributes, and miscellaneous entities such as observations, results or qualifiers. We combined a dedicated lexicon, the knowledge available in rule-based methods [54, 59] adapted to medical Spanish, and state-of-the-art Transformer-based models. Originally developed for clinical trials, the tool is now being released to be used with other medical sub-genres.

## Implementation

The implementation involved several stages. The first steps were creating resources and rules for named entity recognition of the entities of interest. These resources enabled the pre-annotation of a task-specific corpus, which was revised by experts and then used to train deep learning (DL) models in a supervised setting. The MedLexSp lexicon of Spanish medical terms [26] with lemmas and form variants is used for domain entity recognition by employing a dictionary-based approach. The Unified Medical Language System (UMLS) [24] is the main source of domain terminology in MedLexSp. The tool normalizes concepts using SNOMED CT codes [25] or the UMLS concept unique identifiers (CUIs) recorded in MedLexSp.

For temporal entities, we developed rules by adapting HeidelTime [54]. For negation and speculation, we created rules by translating NegEx and ConText [59, 60] and reusing former work for Spanish [86]. Rules for medication-related information were manually developed and refined iteratively in corpus annotation. We also used the lists of administration routes and dosage forms from the Spanish Medicines Agency Nomenclátor [87]. We created rules for the following entity types: Dose, Concentration or Strength; Route or Mode of administration; and Dosage form.

**Fig. 2:**
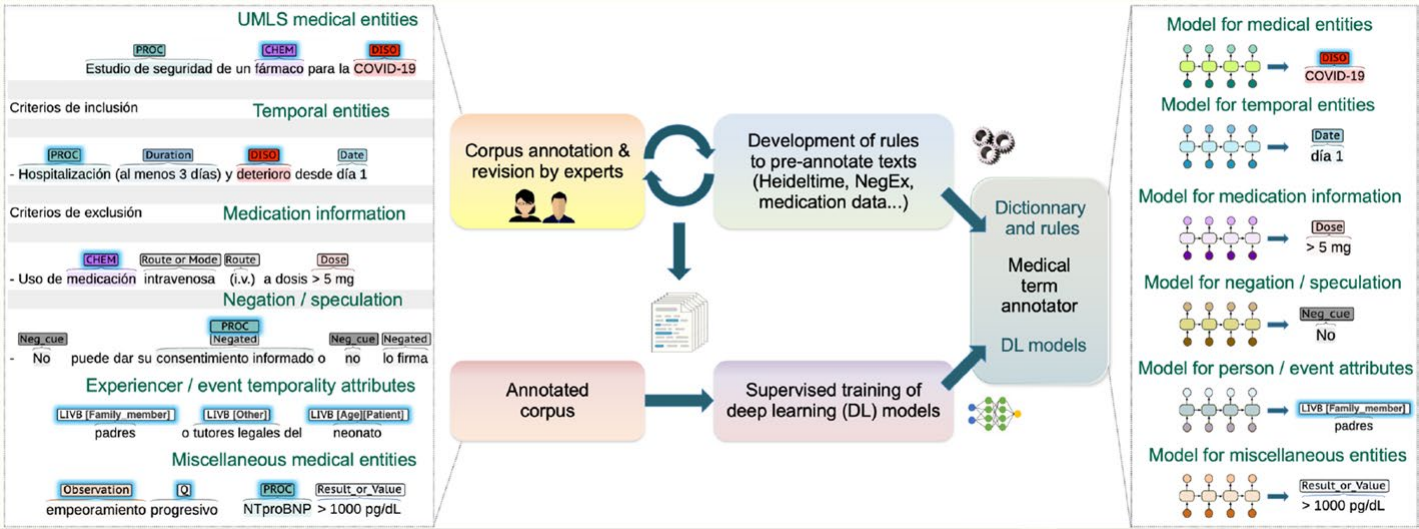
Workflow and system features

The development of rules and the pre-annotation of domain texts were iterative processes: rules were redefined after analyzing the errors of the pre-annotated texts and after being revised by experts, then the improved rules were applied again to new batches of texts to revise, and so on. Figure 2 explains the workflow.

**Fig. 3:**
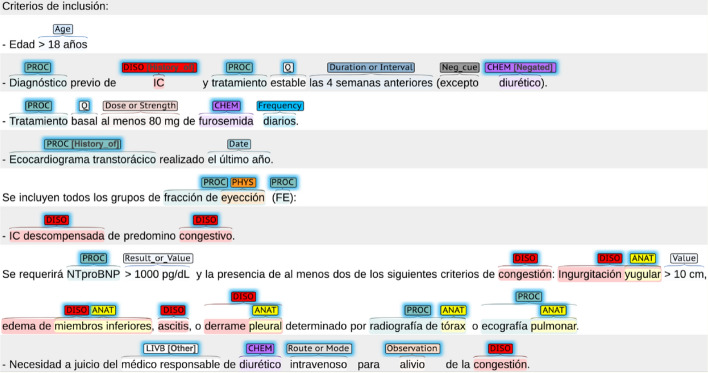
Sample of the annotated corpus used to train the models

To develop the tools, we employed the Clinical Trials for Evidence-Based Medicine in Spanish (CT-EBM-SP) corpus [28]. This is a collection of 1200 texts about clinical trials (292173 tokens). A subset of 500 abstracts come from journals available in PubMed or the Scientific Electronic Library Online (SciELO). Another subset of 700 clinical trial announcements were published in the European Clinical Trials Register. The quality and consistency of the annotations were assessed by computing the inter-annotator agreement (IAA) between annotator pairs, which showed very good agreement (§ Annotation and IAA). The corpus and guidelines are available at the companion repository. Figure 3 is a sample of an annotated text.

We conducted an internal and external validation of the tool, as recommended [88]. The internal evaluation used a held-out test set, i.e. not used in model training. For the external validation, eight medical professionals revised the system annotations on 200 new texts (not used in system development): 100 clinical trial announcements, and also 100 anonymized clinical cases (to assess the performance of other medical sub-genres). The next sections provide more details of each component.

### UMLS entities

In the first version of the corpus [28], only four UMLS semantic groups were annotated: anatomic entities (ANAT; e.g., *arm*), pharmacological and chemical substances (CHEM; e.g., *aspirin*), pathological conditions (DISO; e.g., *diabetes*) and laboratory, diagnostic and therapeutic procedures (PROC; e.g., *radiotherapy*). In the second version (distributed here), we annotated these entity types: medical devices (DEVI; e.g., *probe*), genes and genetic material (GENE; e.g., *BRAF*), physiological processes (PHYS; e.g., *breathing*) and living beings (LIVB, e.g., *patient*; note that this category also includes virus). Nevertheless, we did not use the GENE category to train the models, given the scarce number of instances and the low IAA scores achieved.

### Temporal entities

We followed the TimeML annotation scheme [89] and annotated the following entity types: Date (e.g., *2022*), Duration (e.g., *dos horas*, ‘two hours’), Frequency (called ‘set’ in TimeML; e.g., *semanalmente*, ‘weekly’) and Time (e.g., *noche*, ‘night’). We did not merge entities beginning with *pre-*/*post-* prefixes, as in other annotation projects [90], which mark them as PREPOSTEX. PREPOSTEX generally gathers Date or Time entities that are ambiguous or unspecified (i.e. when there is not enough context to decide if *postoperative* refers to an amount of time expressed in days or hours). However, we kept the distinction between Date and Time in these expressions since it is more valuable for the clinical practice. Finally, we added the entity type Age (e.g., *18 years old*), given that age is an important factor for inclusion or exclusion in clinical trials. In addition, living being entities (LIVB) that also convey age information were annotated with the Age attribute (e.g., *adolescent*, *adults*).

### Medication-related information

We considered the guidelines of previous corpora [66, 91, 92] and marked these types: Dosage form (Form, e.g., *píldora*, ‘pill’), Route or Mode of administration (Route, e.g., *intravenoso*, ‘intravenous’) and Dose, Concentration or Strength (Dose, e.g., *125 mg*). We merged the categories of Dose and Concentration or Strength. This decision might provide coarse-grained results, but seemed more suitable for a general usage of the tool, which can be adapted if needed. Lastly, we annotated Contraindicated in some chemical and pharmacological entities (CHEM) or procedures (PROC). This is typically found in the exclusion criteria of trial announcements (e.g., *Pacientes con contraindicación a corticoesteroides*, ‘Patients with a contraindication to corticosteroids’; the entity *corticosteroids* is Contraindicated).

### Negation and speculation

We marked negation or speculation only on concepts or events. This choice was applied in other biomedical corpora [74, 93–96] and some annotation tools [4, 37]. We did not mark the full negated or speculated scope, as in other works [61, 63, 78, 97]. This criterion can be illustrated with the following sentence: *Los pacientes no habían recibido tratamiento antibiótico* (‘The patients had not receive antibiotic treatment’). The scope of the negation would imply annotating ‘had not receive antibiotic treatment’; however, we only annotated the concept ‘antibiotic treatment’ as Negated. This difference has impacted the performance of the NER models (see § Results). We marked negation and speculation cues (Neg_cue, Spec_cue), and events or entities within the scope of the negation or speculation were marked with Negated or Speculated, respectively. These were mostly marked on UMLS entities (ANAT, CHEM, DEVI, DISO, GENE, LIVB, PHYS and PROC).

### Event temporality and Experiencer

Event temporality attributes are marked on entities referring to procedures and pathological or physiological conditions. These attributes specify whether the event occurred in the past (History_of or Family_History_of), if it will take place in the future (Future) or if it is Hypothetical. Experiencer attributes are only marked on LIVB entities referring to human beings. These attributes indicate if the entity is the experiencer of the condition or procedure (Patient), if it is a patient’s relative (Family_member) or if he/she has another role (Other). We adapted the scheme from previous works [4, 60, 98] to the Spanish language. Importantly, we adopted entity and attribute types used in former annotation projects focused on EHR or clinical case data. This was motivated by the development of a comprehensive annotation framework aimed at improving the mapping of clinical patient data to relevant clinical trials. Nonetheless, we did not use the Hypothetical or Family_History_of attributes to train the models, due to the few instances in the corpus.

### Miscellaneous entities

Lastly, we also annotated a set of clinical entities deemed necessary for the task: CONC (concepts), Food/Drink (*soy*), Observation/Finding (e.g. *relapse*), Quantifier/Qualifier (e.g. *at least 4, severe*) and Result/Value (e.g. *< 3 UNL*).

### Annotation and IAA

We applied the dictionary-based and rule-based tool to clinical trials texts to pre-annotate the data and then revise the output. Three experts annotated the data: a practicing medical doctor with 20 years of medical practice, a medical lexicographer with 18 years of experience in data curation and corpus annotation, and a computational linguist with 18 years of experience in data annotation and natural language processing. To define and learn the annotation guidelines, the experts annotated 12 texts, in several consensus rounds. We used the BRAT annotation tool [27]. After adequate agreement scores were reached, 112 texts were doubly annotated, again with meetings to fix disagreements and refine the guidelines iteratively. In the final step, the leading researcher annotated the remaining texts. Approximately 10% of the corpus (124 texts) was annotated by two or three experts.

We used the F-measure to assess the inter-annotator agreement (IAA) between pairs of doubly annotated sets. The F-measure is considered adequate for contexts where entities can have disparate spans [99]. The IAA values of four UMLS semantic groups (ANAT, CHEM, DISO and PROC) were reported in  [28]. In the first version of the corpus, the IAA scores for these four entity types had an average F1 score of 0.856 (±0.048) in strict match (i.e. the full span and semantic class of the entity must match) after consensus annotations.

**Fig. 4:**
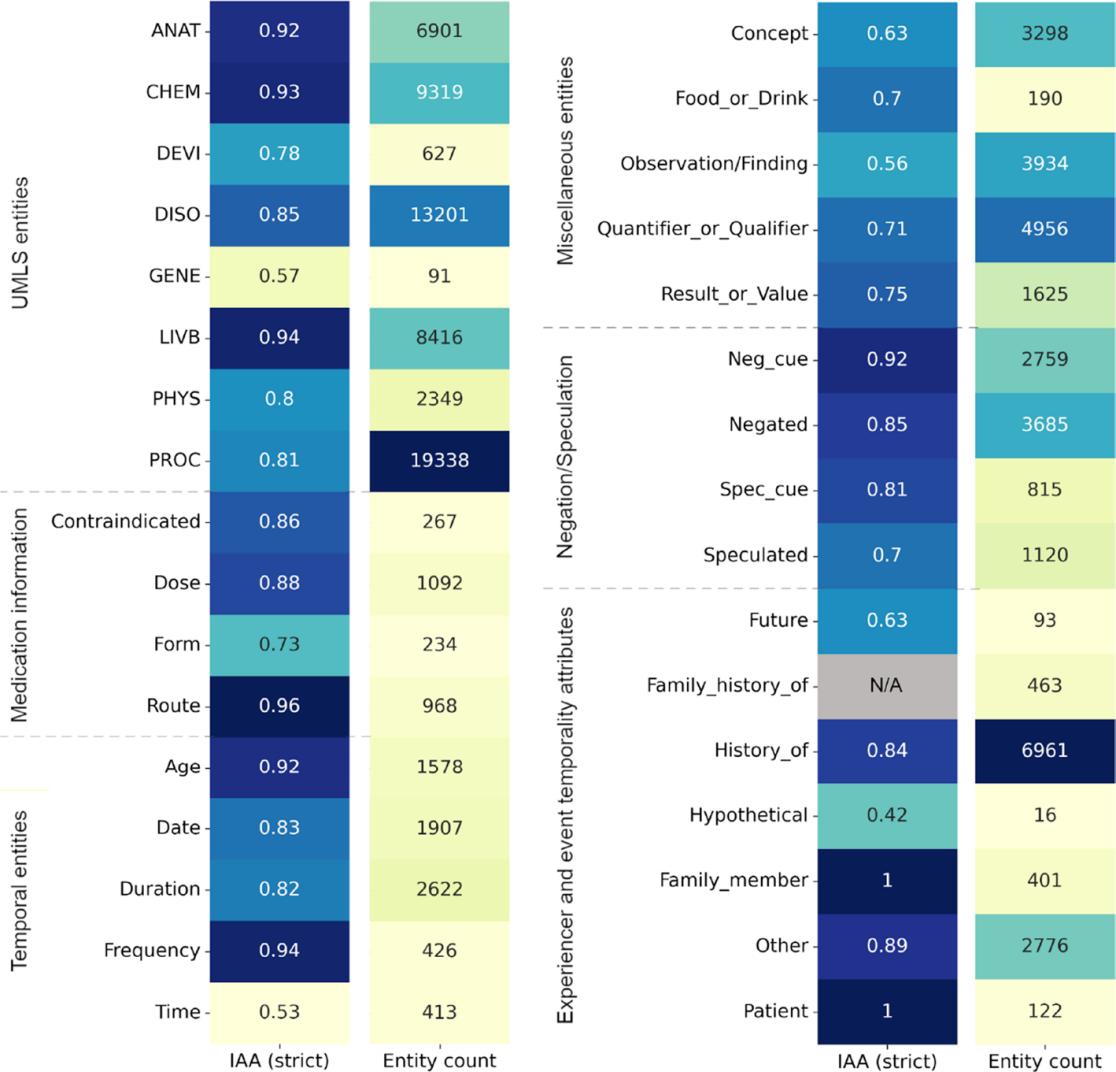
Inter-annotator agreement (IAA) and count of each entity/attribute type

For the new annotated entities, the average IAA was an F1 score of 0.841% (±0.045) with consensus annotations (strict match). For attributes (Age, Negated, Speculated, Contraindicated, Hypothetical, Future, Family_member, Patient and Other), the IAA had an average F1 score of 0.881% (±0.032) with consensus (strict). Note that no IAA was computed for Family_History_of, because no instances appeared in the documents used for double annotation. IAA values were very good, given the large number of different entity types. However, IAA was lower for categories such as Contraindicated, Form, Spec_cue or Speculated, and these had an impact on the lower F1 results obtained with the tested models.

We annotated 86 389 entities (average of 71.99 per text) and 16 590 attributes (average of 13.82 per text). Figure 4 reports the number of annotations per entity type and the IAA scores. For named entity recognition, the annotations were converted to the standard BIO format (B stands for ‘beginning’ of entity; I, ‘Inside’, and O, ‘Out’). The entity attributes were also processed to this format.

### Transformer architecture in BERT models

**Fig. 5:**
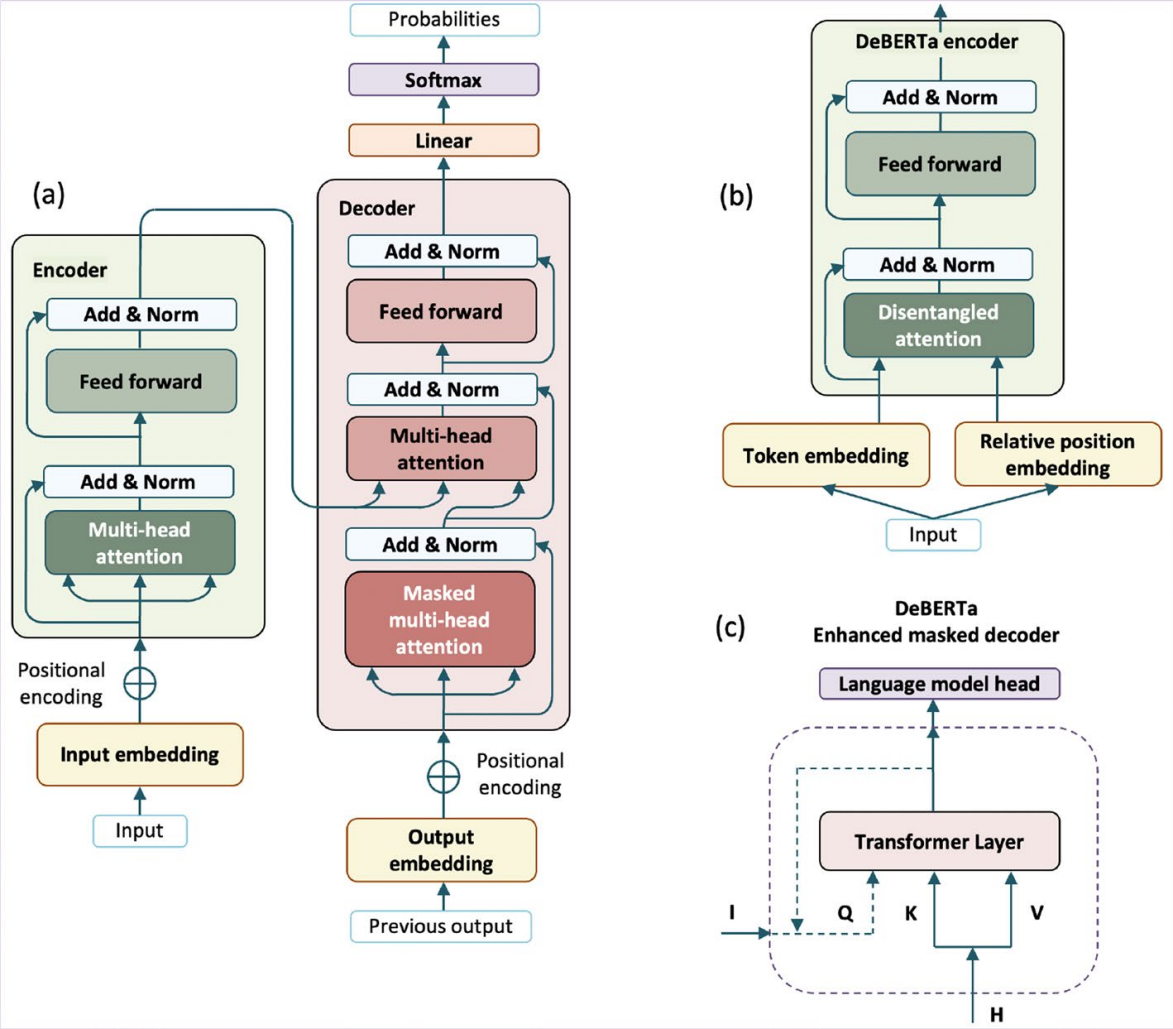
Transformer architecture [100] (**a**); and modified encoder (**b**) and decoder (**c**) in DeBERTa [101]

We used the neural-network based Transformer architecture, as implemented in the Bidirectional Encoder Representations from Transformers (BERT) model [33]. The Transformer architecture replaces the recurrent neural network (e.g., Long Short-Term Memory models) with attention layers [100], and contain an encoder and a decoder (Fig. 5, a). Typically, 6 to 12 encoder layers and 6 to 12 decoder layers are stacked. The encoder converts the input sequence ($x_1, \ldots ,x_n$) into a sequence of continuous representations ($z=(z_1, \ldots ,z_n)$). Encoding layers generate token embeddings and positional embeddings, which bear the information about the location of each word and extract dependencies between them. Note that BERT and RoBERTa also learn representations of segment embeddings. Each layer has a self-attention mechanism that considers the entire context of the sequence, and weighs the importance of each word in relation to others. The multi-head self-attention component employs a set of *attention heads*, which are run in parallel to calculate Query (*Q*), Key (*K*) and Value (*V*) matrices to compute attention scores. The final attention output is passed through a feed-forward neural network with two fully connected layers and ReLU activation in between. A layer normalization is applied, and residual connections are added to the self-attention and the feed-forward components, which help stabilize training. With these *z* representations, the decoder outputs a sequence of symbols ($y_1, \ldots , y_n$). The decoder consists of similar components to those in the encoder; since BERT models do not rely on the decoder for inference, due to space limits, we omit an exhaustive explanation and refer to [100] for more details. Once the sequence passes through all decoder layers, the output is fed into a linear layer followed by a SoftMax activation, which produces probability distributions over the vocabulary to predict the next token.

BERT, and its variants such as RoBERTa [102] or DeBERTa [101], are the most widely-used models with the attention mechanism for sequence labeling tasks. These models’ architecture is composed of a stacked multi-layer bidirectional Transformer encoder, and separate the training in two steps. The first is pre-training a large task-independent language model, with a masked language model objective (i.e. the model has to predict a subset of hidden words). This pre-trained model is, secondly, fine-tuned with data for each task (e.g., named entity recognition). BERT models have an input length limit of 512 tokens at a time (vectors are truncated or padded to that fixed length) and may have 12 or 24 layers.

**Fig. 6:**
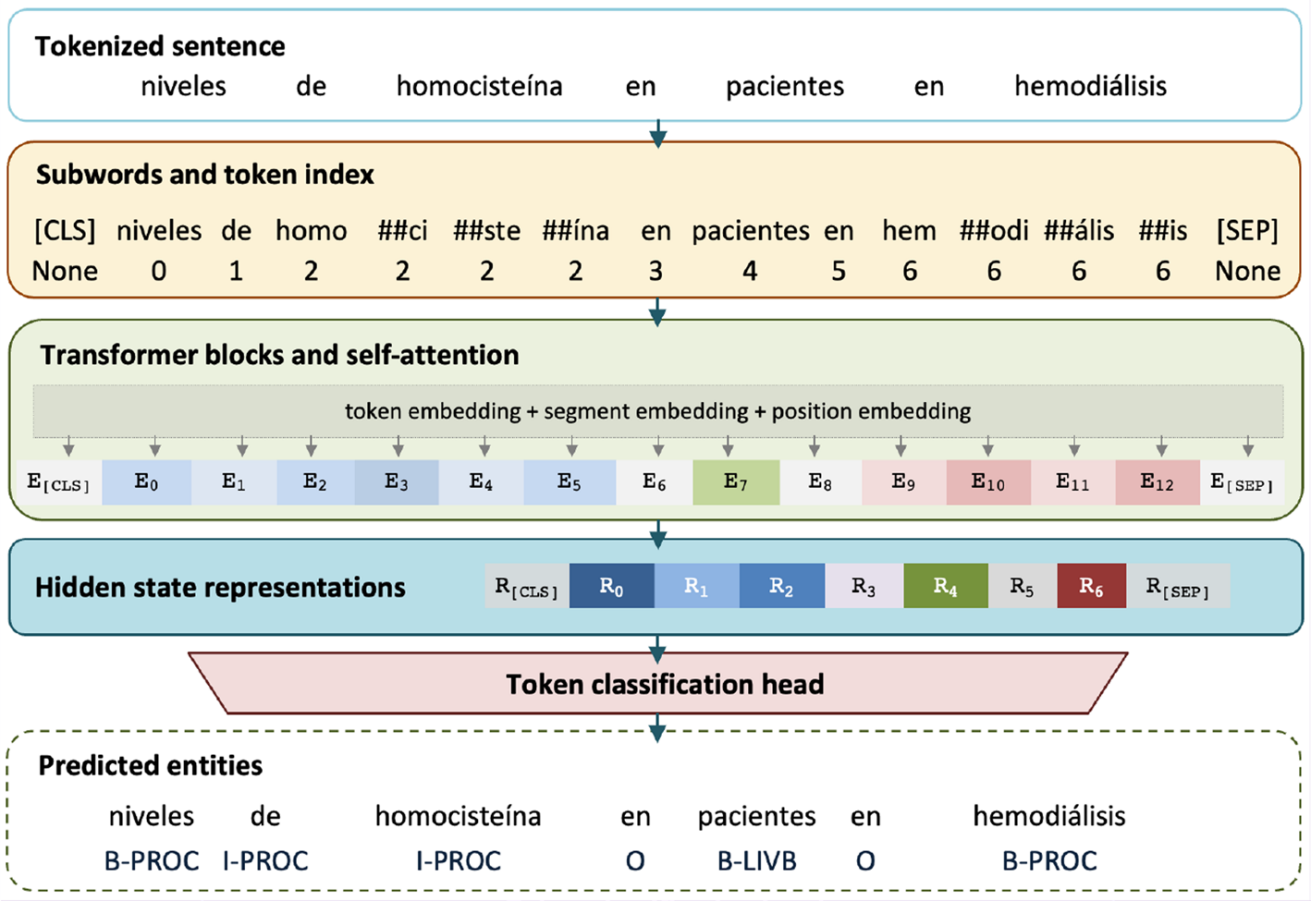
General scheme of Transformer-based medical NER, as implemented in BERT [33]

**Fig. 7:**
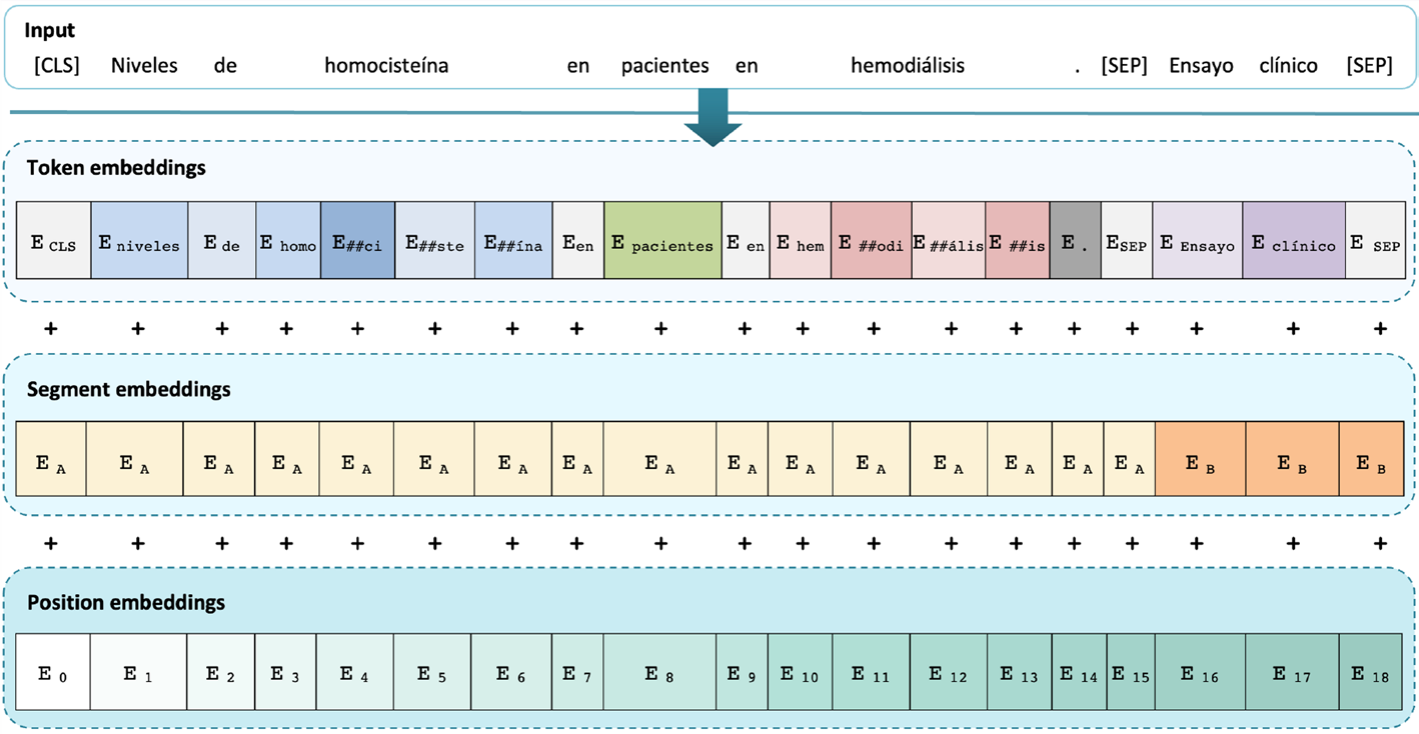
Token, segment and position embeddings in the BERT and RoBERTa Transformer encoder [33]

Figure 6 depicts the Transformer-based processing of a sentence, as implemented in BERT [33] or RoBERTa [102]. First, a [CLS] symbol is added at the beginning of every input sentence (for *classification* purposes) and a [SEP] (*separator*) token is put to separate each segment from the next one. The input text is split into word units (typically, at punctuation signs and white space characters) and sub-word units, using the WordPiece tokenizer [103]. Sub-words are generally used if the original words were not seen in the pre-training corpus, as it occurs in the medical domain: e.g., *homocisteína* (‘homocysteine’) is split in *homo*, *##ci*, *##ste*, *##ína*. Then, a Transformer block processes each sentence element and represents it with token, segment and position embeddings (Fig. 7 depicts this process for BERT and RoBERTa models). The segment embedding is a value corresponding to the sentence or phrase where each token occurs, separated by the [SEP] symbol. In Fig. 7, two segments appear: *niveles de homocisteína en pacientes en hemodiálisis* (‘homocysteine levels in hemodialysis patients’, segment A) and *Ensayo clínico* (‘Clinical trial’, segment B). Every token in the same segment has the same segment embedding vector. The input representation is combined by summing the token, segment and position embeddings to obtain a hidden representation with contextual information; note that DeBERTA models encode the input with two vectors (for token and relative position), as explained in the next section. Lastly, a token classification head for named entity recognition—made up of a dense layer and a SoftMax activation—outputs the semantic class and span of the named entities.

### Training of models

The annotated corpus was used in a supervised setting to train Transformer-based DL models. We fine-tuned six model variants for: (1) UMLS semantic groups; (2) medication information; (3) temporal entities; (4) negation and speculation; (5) miscellaneous medical entities; and (6) experiencer and temporality attributes.

First, we tested domain-specific RoBERTa-based [102] pre-trained models available for medical Spanish. RoBERTa (Robustly Optimized BERT Pre-training Approach) is a model variant that was pre-trained for longer (125000 additional steps), with a larger batch size (8000 samples per batch), using dynamic masking, and on a larger dataset (160 gigabytes) than the collection used to train BERT. RoBERTa also removes the Next Sentence Prediction task used for pre-training BERT, which makes it a more optimized model. We tested the bsc-bio-ehr-es model, which further pre-trained RoBERTa with medical texts in Spanish from SciELO, Wikipedia, EMEA or PubMed, and also with clinical data from EHRs [49]. This model uses the Byte-Pair Encoding (BPE) tokenizer, which is more efficient for subword tokenization. To analyze the influence of bilingual, domain pre-training data, we also tested EriBERTa [104] (base version), a bilingual RoBERTa-based bilingual model. EriBERTa base was pre-trained on an English and Spanish corpus. The English medical pre-training data include texts from EMEA, PubMed and ClinicalTrials.gov; and subsets of the Spanish collection come from SciELO, EMEA, PubMed, SNOMED CT and a Spanish clinical cases corpus. Lastly, we compared both models to the RoBERTa XLM Spanish Clinical model (hereafter, CLIN-X-ES) [50] large version. CLIN-X-ES is derived from the XML RoBERTA multilingual model (originally pre-trained on 2.5 terabytes of the CommonCrawl corpus for 100 languages), by continuous pre-training on a corpus of medical texts from SciELO, MedlinePlus, EMEA or PubMed. This model implements the XLM-R tokenizer and computes the input on subword level (not on word level); also, the cross-sentence context is incorporated in the input. Lastly, a Conditional Random Field (CRF) layer computes the output.

Second, we assessed multilingual, general-domain models, to examine further whether the difference in performance across models might be related to domain-specific or multilingual pre-training data. We evaluated the multilingual BERT (from here on, mBERT) base model, which was pre-trained on a corpus derived from Wikipedia in 104 languages. We also tested the Decoding-enhanced BERT with Disentangled Attention (DeBERTa) model, which presents two innovations in the BERT architecture. First, DeBERTa features a disentangled attention mechanism: each word is represented by separate vectors (not only by one sum vector, as in the original BERT) to encode the content and relative position separately (Fig. 5 b). With this approach, the self-attention layer models the dependency between near tokens in a better way. Second, DeBERTa features an enhanced mask decoder for pre-training (Fig. 5 c; *Q* stands for ‘Query’; *K*, for ‘Key’; *V*, for ‘Value’; and *I*, for ‘Input’; and *H*, for ‘Hidden state’). The enhanced mask encoder employs the information about the content and the position for masked language modeling. Information about absolute positions is introduced in the decoding layer (before the SoftMax layer) to predict masked tokens during pre-training. By means of this technique, the model gives more importance to the absolute position of words. In our experiments, we specifically used the multilingual DeBERTa (hereafter, mDeBERTa) model vs 3 [101] base version, which is a multilingual, general-domain model pre-trained on the CC-100 corpus (CommonCrawl for over 100 languages).

Table 1 summarizes the characteristics of the Transformer-based models we tested and the pre-training details. To fine-tune and release the models, we used Transformers Hugging Face [32], which facilitates the reuse and adaptation of available models, and the replication of experiments. We used the AutoModelForTokenClassification class for NER. All the tested models are shared at the Hugging Face hub.

**Table 1:** Characteristics of the Transformer-based models and pre-training details (medical-domain models are italized in rows 2-4)

Model	PT corpus size	#A	#H	#L	#P	#V
RoBERTa EHR (**bsc-bio-ehr-es**)	>1B tok	12	768	12	125M	52K
EriBERTa (**EriBERTa-base**)	900M tok	12	768	12	125M	50K
CLIN-X-ES (**xlm-roberta-large-spanish-clinical**)	790MB	16	1024	24	550M	250K
mBERT (bert-base-multilingual-cased)	2.5T	12	768	12	110M	110K
mDeBERTa (mdeberta-v3-base)	2.5T	12	768	12	190M	250K

*A*: attention heads; *B*: billion; *H*: hidden size; *K*: thousand; *L*: number of layers; *M*: million;

*MB*: megabytes; *P*: parameters; *PT*: pre-training; *T*: terabytes; *Tok*: tokens; *V*: vocabulary size

As baselines for comparison, we applied the rules we developed for temporal annotation and medication information extraction. We also tested the annotated corpus on a Bidirectional Long Short-Term Memory (Bi-LSTM) architecture with Conditional Random Fields (CRF) for sequence labeling. We used the implementation in the FLAIR framework, which models the linguistic context through *contextual embeddings* [34], but does not include any local nor global attention mechanism [105, 106]. The FLAIR implementation has one bidirectional LSTM layer with 1024 hidden states each (totaling 2048, 1024 for each direction).

To select the best configurations, we used the train (60%) and development splits (20%) from  [28] (respectively, 720 and 240 texts). Once the best models were obtained, we tested then on a held-out set (20%, 240 texts). Because preliminary experiments for negation and speculation yielded poor results, we also used the NUBEs corpus [61] in our data. We used train/dev/test splits and added them to the corresponding splits in our corpus. For the negation/speculation model, we manually converted the format and labels from the NUBEs corpus in order to fit our criteria of labeling negation and speculation (e.g., label names were changed).

Table 2 describes the data splits used to train, validate and test the models (in the internal evaluation and in the human evaluation, *HE*); we include the count of tokens and texts in the training data enriched with the NUBEs corpus (+518068 tokens). We also count the subsets of augmented data for applying the RoBERTa EHR and the CLIN-X-ES models to clinical cases (§Extending the models to clinical cases). These augmented data contain 100 clinical cases (*CC*, +29808 tokens) and summaries of product characteristics (SPCs, +51057 tokens).

**Table 2:** Data splits to train and test each model (listed in the header)

	UMLS entities, temporal entities, miscellaneous entities, temporality/experiencer attributes		Medication information		Negation/speculation	
	#Texts	#Tokens	#Texts	#Tokens	#Texts	#Tokens
Train	720	175203	720	175203	7739	693271
Train (CC)	820	205011	1085	285876	7839	723079
Dev	240	58670	240	58670	240	58670
Test	240	58300	240	58300	240	58300
Test (HE)	200	27332	200	27332	200	27332

*CC*: clinical cases; *HE*: human evaluation

**Table 3:** Fine-tuning hyperparameters of the tested models

Model	B	Mx Ep	LR	Optim	Pat	Seed
Bi-LSTM-CRF (FLAIR)	16	100	0.1	SGD	5	Random
RoBERTa, EriBERTa,	16	20	2e-05	Adam	5	{100, 200, 300, 400, 500}
mBERT and mDeBERTA vs 3						
CLIN-X-ES	8	30	2e-05	Adam	5	{100, 200, 300, 400, 500}

*B*: ‘batch’; *LR*: ‘fine-tune learning rate’; *Mx Ep*: ‘maximum number of epochs’; *Pat*: ‘Patience’;

*Optim*: ‘Optimizer’; *SGD*: ‘stochastic gradient descent’

We used a Zotac Geforce RTX 3090 GPU of 24 GB RAM for the experiments. The models were trained with early stop with a patience of 5 (i.e. the training stopped if the F1 score did not improve after 5 epochs). Table 3 shows the hyperparameters.

### Annotation pipeline

Given an unstructured medical text (e.g., a clinical trial announcement or a clinical case), stage 1 involves pre-processing (e.g., changing characters that cause problems to the Transformer-based models), sentence splitting, tokenization and part-of-speech tagging by means of spaCy or Stanza (we used spaCy in this work). Then, the UMLS medical entities are detected with the dictionary (MedLexSp) [26] or with a BERT-based model. A list of exceptions can be applied to the output. For example, a specific semantic group can be excluded if it is unnecessary for a task: e.g. ACTI (activity entities), which are recognized only with the dictionary. Stage 2 involves named entity recognition of temporal entities, medication information, miscellaneous clinical entities, and attributes expressing negation/speculation or temporality/experiencer. This can be customized according to the user’s needs (the output of stage 1 can be passed to stage 3 straightaway) and is processed sequentially with dedicated models. Lastly, stage 3 converts the output to JSON or BRAT format. Figure C.17 (Appendix) outlines the steps.

### Extending the models to clinical cases

To test the generalizability of the trained models to other medical text types, we used 200 clinical cases already anonymized and prepared by medical residents or under a Creative Commons License.^1^ We utilized 100 texts (comprising 29808 tokens) to further fine-tune the RoBERTa EHR and CLIN-X-ES models and adapt them to the new subdomain; and 100 texts (27332 tokens) for a human evaluation by medical professionals.

For the medication information model applied to clinical cases, we also included in the fine-tuning data 265 text samples (51057 tokens) of summaries of product characteristics (SPCs) from the Spanish Drug Information Center (CIMA). We selected SPCs from a subset of drugs included in the WHO Model List of Essential Medicines [107]. We used excerpts from sections corresponding to the Medication name, Dosage form, Administration mode and Contraindications. We also applied a data-augmentation technique for synonym replacement [108] using UMLS Concept Unique Identifiers available in MedLexSp [26]. For example, full forms (*intravenoso*, ‘intravenous’) were replaced with abbreviations/acronyms (*IV*). Some contexts required revision to fix gender agreement and replacement errors. In summary, we trained the models for medication information entities with the CT-EBM-SP corpus, 100 clinical cases and 265 text samples from SPCs (Table 2).

After inspecting the output of the models, we found that the RoBERTa EHR models were the most suitable for integration into the annotation pipeline of the medical entity recognizer (see §Results); accordingly, medical professionals evaluated only these models. We did not fine-tune the EriBERTa, mBERT or mDeBERTa models with the 100 clinical cases nor with the SPCs for the current version of the tool.

### Evaluation

#### Internal validation

We computed precision (P, or positive predictive value), recall (R, or sensitivity) and F1 with the seqeval library [109]:

$$\begin{aligned} P = \frac{TP}{TP + FP} \qquad R = \frac{TP}{TP + FN} \qquad F1 = \frac{ 2 P R }{ P + R } \end{aligned}$$

(*TP*: true positives; *FP*: false positives; *FN*: false negatives). We report micro-average scores because of the classification imbalance: e.g., anatomic entities (ANAT) or devices (DEVI) are scarce compared to chemical instances (CHEM). Results are reported on exact match at named-entity level (i.e. a true positive is counted if the models’ prediction and reference match in scope and class). We provide the average and standard deviation of five experimental rounds with different initialization seeds. Carbon emissions of the models were estimated with a calculator online [110].

**Table 4:** Comparison models (average ± standard deviation); medical-domain models are italized;general models are underlined; best results in bold (*P*: ‘precision’; *R*: ‘recall’)

	UMLS entities			Negation / speculation		
	P	R	F1	P	R	F1
Bi-LSTM-CRF	0.815	0.813	0.814	0.845	0.776	0.809
	(± 0.003)	(± 0.002)	(± 0.002)	(± 0.002)	(± 0.006)	(± 0.004)
mBERT	0.875	0.887	0.881	0.855	0.852	0.853
	(± 0.004)	(± 0.005)	(± 0.001)	(± 0.006)	(± 0.010)	(± 0.004)
*RoBERTa EHR*	0.878	0.894	0.886	0.855	0.864	0.859
	(± 0.003)	(± 0.003)	(± 0.002)	(± 0.005)	(± 0.009)	(± 0.006)
*EriBERTa*	0.881	0.896	0.889	0.861	0.871	0.866
	(± 0.005)	(± 0.002)	(± 0.003)	(± 0.008)	(± 0.005)	(± 0.006)
*CLIN-X-ES*	**0.906**	0.911	0.909	**0.871**	**0.874**	**0.873**
	(± **0.005**)	(± 0.008)	(± 0.003)	(± **0.008**)	(± **0.007**)	(± **0.005**)
mDeBERTa	0.904	**0.916**	**0.910**	0.861	0.871	0.866
	(± 0.001)	(± **0.003**)	(± **0.002**)	(± 0.009)	(± 0.009)	(± 0.006)
	Temporal entities			Medication information		
	P	R	F1	P	R	F1
Rules	0.824	0.841	0.833	0.628	0.786	0.698
Bi-LSTM-CRF	0.899	0.859	0.879	0.895	0.740	0.810
	(± 0.007)	(± 0.005)	(± 0.006)	(± 0.002)	(± 0.015)	(± 0.009)
mBERT	0.874	0.862	0.868	0.856	0.823	0.839
	(± 0.007)	(± .006)	(± 0.004)	(± 0.007)	(± 0.011)	(± 0.004)
RoBERTa EHR	0.900	0.900	0.900	0.873	0.871	0.872
	(± 0.011)	(± 0.007)	(± 0.001)	(± 0.022)	(± 0.015)	(± 0.004)
EriBERTa	**0.922**	0.908	**0.915**	0.874	0.828	0.850
	(± **0.004**)	(± 0.011)	(± ** 0.007**)	(± 0.024)	(± 0.007)	(± 0.010)
CLIN-X-ES	0.899	0.895	0.897	0.883	0.863	0.872
	(± 0.017)	(± 0.005)	(± 0.010)	(± 0.021)	(± 0.015)	(± 0.008)
mDeBERTa	0.909	**0.918**	0.913	**0.897**	**0.885**	**0.891**
	(± 0.009)	**(± 0.006)**	(± 0.005)	**(± 0.012)**	**(± 0.014)**	**(± 0.008)**
	Miscellaneous entities			Experiencer/Temporality attributes		
	P	R	F1	P	R	F1
Bi-LSTM-CRF	**0.721**	0.536	0.613	0.891	0.816	0.852
	(± **0.006**)	(± 0.005)	(± 0.004)	(± 0.007)	(± 0.004)	(± 0.001)
mBERT	0.674	0.623	0.647	0.868	0.831	0.849
	(± 0.027)	(± 0.015)	(± 0.006)	(± 0.023)	(± 0.014)	(± 0.005)
RoBERTa EHR	0.685	0.669	0.677	0.877	0.835	0.856
	(± 0.008)	(± 0.004)	(± 0.003)	(± 0.009)	(± 0.008)	(± 0.006)
EriBERTa	0.703	0.666	0.684	0.890	0.848	0.868
	(± 0.017)	(± 0.009)	(± 0.006)	(± 0.007)	(± 0.008)	(± 0.002)
CLIN-X-ES	0.715	**0.672**	**0.692**	0.894	0.863	0.878
	(± 0.014)	(± **0.016**)	(± **0.007**)	(±0.013)	(± 0.010)	(± 0.005)
mDeBERTa	0.702	0.670	0.686	**0.898**	**0.882**	**0.890**
	(± 0.011)	(± 0.007)	(± 0.004)	**(± 0.009)**	**(± 0.008)**	**(± 0.005)**

#### External validation (human evaluation)

As a use case in a real setting, eight medical professionals revised the annotations obtained with the tool (using the RoBERTa EHR models) on 200 new texts not employed for system development. First, 100 texts (29851 tokens) announcing trials published in 2022-23 were downloaded from EudraCT after developing the system. Second, to test the generalizability to other medical sub-genres, the evaluators checked the annotation of 100 clinical cases with a Creative Commons license (27332 tokens). Three medical doctors, four medical interns and one nurse participated; their average age was 33.0 years and the average years of medical practice was 6.88 years. Evaluators were instructed about the entity types and scope. The evaluation texts are available at the companion repository. Participation was remunerated afterwards.

## Results

### Results of the internal validation

Table 4 shows the results of the compared methods. Rules for medication information extraction and temporal entity recognition performed below the neural-network-based models, although the recall for the medication information entities (R = 0.786) was higher than that of the Bi-LSTM-CRF model (R = 0.740). For their part, Transformer-based models showed better performance compared to the Bi-LSTM-CRF model across all subtasks. The RoBERTa EHR and EriBERTa models performed similarly well, the latter tending to achieve slightly higher F1 scores. When comparing the performance of multilingual general-domain models, mBERT tended to yield lower scores than the RoBERTa-based models, but the mDeBERTa vs 3 model outperformed the other models for all tasks. However, the CLIN-X-ES models exhibited comparable performance, and outperformed the rest of RoBERTa-based models, excepting for the recognition of temporal entities. In Appendix B, Figure B.8 plots the F1 measure per model (averaged over 5 experimental runs); and Table B.8 presents the results per entity type of the RoBERTa EHR models, which were used in the human evaluation.

Regarding the training time of the clinical trial texts, the RoBERTA EHR model needed fewer training epochs; the estimated carbon footprint of each experimental round for the RoBERTa EHR models was of 63 g (eq. CO$_{2}$) per model (this value and the following are the average of 5 rounds). The EriBERTa models required a similar number of training epochs, and given their shorter training times, they had an estimated carbon footprint values of 33 g (eq. CO$_{2}$) per model. The CLIN-X-ES models also needed similar training epochs, but each one had longer training times; their estimated carbon footprint was of 209 g (eq. CO$_{2}$) per model. The mBERT and mDeBERTa models showed a similar trend, with fewer training epochs (although each epoch in mDeBERTa was longer). The estimated carbon footprint of each mBERT model was of 62 g (eq. CO$_{2}$), and the estimated carbon footprint of each mDeBERTa model was of 115 g (eq. CO$_{2}$). Lastly, the Bi-LSTM-CRF models needed more training epochs and longer training times; their estimated carbon footprint was of 263 g (eq. CO$_{2}$) per model. Table A.6 in Appendix A includes all the details.

With regard to the annotation times, we compared both RoBERTa-based models using the 100 new EudraCT texts and the 100 clinical cases for the human evaluation. Speed of processing was measured in a laptop with a CPU Intel Core i7 processor (2.8 GHz), 16 GB of RAM and Mac OS. Table A.7 in Appendix A shows that the RoBERTa EHR and EriBERTa models were faster compared to and mDeBERTa and CLIN-X-ES; we did not compare them with the Bi-LSTM or the mBERT models, given the worse performance of the latter models.

We inspected the errors made by these models on a subset of the test split (§Discussion). The CLIN-X-ES models made critical errors in medication and temporal information, and the mDeBERTa models did not label several medical abbreviations and acronyms. Moreover, these models had longer annotation times and a higher environmental impact of fine-tuning. We thus selected the RoBERTa EHR models to be evaluated by medical professionals (note that the EriBERTa model was publicly released after the human evaluation has conducted). In the current version of the medical entity recognition tool, we integrated the RoBERTa EHR models; however, we release the rest of fine-tuned models in the Hugging Face hub.

### Results of the human evaluation

Table 5 shows the comparison between the evaluators’ revisions and the system output of 100 new texts from EudraCT and 100 clinical cases. The average F1 score (strict match) of the tool was of 0.858 (± 0.032) on the 100 trial announcements, and of 0.910 (± 0.019) on the 100 clinical cases. Interestingly, performance on the 100 cases was higher than on the 100 new EudraCT texts. The fact that the RoBERTa EHR model was trained on clinical data may account for this outcome.

**Table 5:** Comparison of system predictions and human evaluation of 100 new trials and 100 clinical cases

	Strict			Relaxed		
	P	R	F1	P	R	F1
EudraCT						
UMLS entities	0.908	0.879	0.893	0.965	0.928	0.946
	(± 0.037)	(± 0.049)	(± 0.042)	(± 0.018)	(± 0.029)	(± 0.020)
Temporal entities	0.901	0.899	0.899	0.933	0.933	0.931
	(± 0.098)	(± 0.086)	(± 0.085)	(± 0.062)	(± 0.066)	(± 0.049)
Medication information	0.838	0.793	0.803	0.934	0.879	0.893
	(± 0.155)	(± 0.158)	(± 0.140)	(± 0.122)	(± 0.161)	(± 0.112)
Negation / speculation	0.785	0.819	0.795	0.892	0.912	0.899
	(± 0.153)	(± 0.095)	(± 0.125)	(± 0.126)	(± 0.056)	(± 0.090)
Miscellaneous entities	0.801	0.714	0.754	0.872	0.776	0.820
	(± 0.075)	(± 0.073)	(± 0.068)	(± 0.064)	(± 0.061)	(± 0.055)
Experiencer / temporality	0.897	0.854	0.873	0.939	0.908	0.918
	(± 0.069)	(± 0.093)	(± 0.074)	(± 0.113)	(± 0.091)	(± 0.093)
Overall	0.876	0.842	0.858	0.942	0.897	0.919
	(± 0.029)	(± 0.039)	(± 0.032)	(± 0.020)	(± 0.028)	(± 0.019)
		**Clinical cases**				
UMLS entities	0.946	0.929	0.937	0.971	0.949	0. 960
	(± 0.027)	(± 0.023)	(± 0.024)	(± 0.020)	(± 0.014)	(± 0.015)
Temporal entities	0.955	0.952	0.953	0.988	0.986	0.987
	(± 0.063)	(± 0.056)	(± 0.059)	(± 0.018)	(± 0.016)	(± 0.014)
Medication information	0.948	0.945	0.946	0.989	0.990	0.989
	(± 0.905)	(± 0.104)	(± 0.096)	(± 0.033)	(± 0.033)	(± 0.027)
Negation / speculation	0.938	0.968	0.952	0.962	0.992	0.976
	(± 0.063)	(± 0.031)	(± 0.042)	(± 0.059)	(± 0.014)	(± 0.034)
Miscellaneous entities	0.757	0.878	0.812	0.796	0.933	0.858
	(± 0.085)	(± 0.060)	(± 0.072)	(± 0.068)	(± 0.033)	(± 0.048)
Experiencer / temporality	0.759	0.880	0.811	0.803	0.932	0.859
	(± 0.126)	(± 0.110)	(± 0.106)	(± 0.117)	(± 0.094)	(± 0.092)
Overall	0.896	0.924	0. 910	0.926	0.955	0.940
	(± 0.023)	(± 0.018)	(± 0.019)	(± 0.018)	(± 0.010)	(± 0.012)

System predictions regarding the seven UMLS entity types and the temporal annotations were in line with the results obtained in our held-out test set. In the 100 new EudraCT trials, results for medication information and negation or speculation were moderately below our scores in the test set; but note that their standard deviation scores were higher. The system performed very well in some texts, but not in other ones. A similar trend is observed in models for miscellaneous medical entities and experiencer and temporality attributes. The models for medication information, negation and speculation, miscellaneous medical entities, and experiencer and temporality attributes seem to vary widely in a real-world setting.

Upon analyzing the discrepancies between the system’s predictions and the human evaluations, we observed that the tool failed to annotate certain Route entities that were missing in the training data (e.g., *systemic*, which caused inconsistencies across annotators). Negation and speculation caused several misinterpretations regarding the scope of Negated or Speculated. In particular, contexts involving laboratory tests, or where the scope is unclear, were often ambiguous for human evaluators. Several mismatches affected the PHYS and Observation categories, especially in entities expressing normal findings (e.g., *normoperfundido*, ‘normal perfusion’), and generally in the 100 clinical cases. The PROC semantic group also caused ambiguities in diagnostic tools or scales (e.g., *ECOG*) or observable entities that can be interpreted as an observation (e.g., *left ventricular ejection fraction*).

The medical professionals also gave us some feedback. Some aspects revolved around the scope of the annotation, which needed discontinuous entity marking (e.g., *pregnant or lactating patients*). However, discontinuous annotations are currently not supported in this version of the tool.

## Discussion

Overall, Transformer-based models achieved the highest scores compared to our manually-crafted rules or the Bi-LSTM-CRF framework. In the comparison between the RoBERTa-based models, we observed that a bilingual, domain-specific model (EriBERTa) slightly surpassed the monolingual medical model RoBERTa EHR (except for the recognition of medication information). This suggests that cross-lingual transfer learning has a positive impact. Indeed, CLIN-X-ES outperformed both models, and a plausible explanation might be that it was pre-trained on the multilingual XLM RoBERTa model and further pre-trained on Spanish medical corpora. Interestingly, we identified contradictory findings when comparing multilingual general models to monolingual domain models. Although mBERT tended to show a lower performance, the mDeBERTa model generally outperformed RoBERTa ERH and CLIN-X-ES, which were both trained with medical data. One explanation could be the specific architecture of the mDeBERTa model, namely the disentangled attention mechanism and the use of absolute position information, which enables the model to learn better dependencies between words. To confirm whether this performance is due to the multilingual pre-training data or to the model architecture, a medical mDeBERTa model should also be tested in our data, which is, however, currently unavailable. Nonetheless, the mDeBERTa and CLIN-X-ES models did not extract key medical entities, abbreviations and acronyms for our task; therefore, we used the RoBERTa ERH models in the external evaluation by medical professionals.

We analyzed the errors in the output of the RoBERTa EHR model for the human evaluation. Figure B.9 in Appendix B includes a detailed error analysis based on an standard taxonomy [111]; Figures B.10-B.15 include examples; and Figure B.16 shows the confusion matrices of predicted and gold standard labels per category. Many errors involved the scope of DISO, PROC, Duration, Observation, Negated or Speculated entities. These occurred when the model labeled modifiers as part of the entity (e.g., *small* *children*), or vice versa. False negatives occurred in classes with scarce instances in the corpus (ANAT, DEVI, PHYS, Contraindicated, Form, Food or Future, and lexical negation/speculation cues). Proper names and acronyms caused both false positives (e.g., *dialyzer* *AN69ST*$^{{\underline{{\circledR }}}}$) and false negatives (*GE*, ‘experimental group’). The semantic class was often wrong between CHEM and PROC, Date and Time, DISO and Observation, or Negated and Speculated.

This analysis revealed that a contributing factor to the observed errors is the insufficient number of annotated samples for specific categories within the training dataset. Another source of errors occurred under the zero-shot setting, when unseen instances in the training data (e.g., acronyms or brand names) were unannotated or misclassified. These would be the major weaknesses of the current version of the tool, especially if false negative errors cause valuable information loss. Errors affecting the scope of entities seem to arise owing to ambiguous contexts, in particular of negated and speculated phrases. Nonetheless, human evaluators did not find severe errors in this respect; and in the case of multi-word entities, missing tokens might be considered a minor error if the entity head is labeled (e.g. *gastric bleeding* vs. *upper gastric bleeding*). The error analysis also provided us with insights into future improvements. To alleviate the false negative and zero-shot issues, we would need to conduct more annotations—specifically for the infrequent classes—and train the models with enriched annotated data. To improve the detection of negation and speculation, we could try methods based on syntax [112] or data augmentation [113].

We next present a comparative analysis of our results with those reported in other studies for similar tasks. We refer to recent works for a comparison of systems on EHRs [114] and an analysis of encoder-based models on clinical datasets [115]. Table D.9 in Appendix D includes specific figures reported by the authors, with an exact match criterion, excepting the work by [67], who reported relaxed-match results. Replicating other teams’ results was out of the scope of this work.

In general, direct comparison of our results is not feasible due to the substantial variability among annotation schemes. We used a subset of UMLS semantic groups; however, the UMLS subset of cTAKES differs from our scheme (e.g., we did not separate signs/symptoms and disorders into different categories). For English clinical trial data, the UMLS was not used [116–118]; and Criteria2Query [5] was evaluated on the OMOP scheme [119].

Regarding temporal entities, our results with a Transformer model are slightly higher than those reported by other teams. However, we used the Age class and the TimeML scheme. Other groups restricted to the four categories of TimeML and achieved competitive results using rule-based methods (generally based on HeidelTime [120, 121]), machine-learning (as implemented in the cTAKES’ temporal expression extractor [55]) and neural models [82, 83].

Our annotation scheme also differs from other studies regarding drug-related entities. We merged Dose and Strength, and did not include classes such as Condition or Reason, considered in other works  [66, 92]. Results cannot be compared to those by other teams who applied rule-based methods [122], deep learning-based algorithms  [66] or ensemble methods [67]. Nonetheless, to the best of our knowledge, no similar work has been done on medication data in Spanish texts using deep learning.

Regarding negation and speculation, we annotated this type of information exclusively for concepts or events, which is similar to the cTAKES approach [4]. Although we did not annotate the scope of negation or speculation, most state-of-the-art works achieved similar outcomes. Recognizing cues generally achieves higher scores compared to scopes, and this is consistent across medical reports [61, 64], medical literature [62] or clinical trials [5]. This trend is also observed with neural-network-based approaches in Spanish, French or Brazilian Portuguese [63]. We also obtained higher scores recognizing negation rather than speculation.

As for event temporality attributes (History_of, Family_History_of and Future), comparison with other studies is challenging, as these classes are frequently merged into more general categories. Some examples are status attributes in [4] and historical attributes in ConText [60]. Few projects considered experiencer attributes (Patient, Other and Family_member), but consistent with our findings, these generally demonstrated high scores.

Miscellaneous medical entities show a broader variability in results. Observations or Findings did not achieve the highest scores, which is consistent with the results reported by other teams [77, 95, 123]. This class can be confused with disorders or results, and models tend to perform poorly. The same trend occurs in quantifier/qualifier entities, which may include a wide range of adjectives or expressions that other schemes represent with more specific classes: e.g. Multiplier [117] or Modifier [123]. In contrast, entities expressing results or values have high scores.

### Limitations

Among our limitations, we only used texts about clinical trials to develop the tool. We trained new models with 100 anonymized clinical cases and tested them on 100 different cases, but we need to confirm the performance for other text genres, especially real EHRs. Moreover, our annotation scheme might be coarse for some tasks. The DISO category merged signs, symptoms and disorders; and Dose includes both dosage and concentration or strength. We largely depend on the UMLS categories, which might be inadequate for other tasks, and the tool is not compatible with other standards, e.g., OHDSI OMOP [119]. However, the tool can be used to pre-annotate texts using labels that can be adapted to specific tasks. Further improvement is required in the recognition of drug-related information; achieving this will necessitate additional annotated data. Discontinuous entities (i.e. separated by non-annotated tokens) were discarded to train the models: e.g., *vacuna anual* *contra la gripe* (‘influenza virus [..] vaccine’). Furthermore, new architectures and language models need to be tested. Lastly, although the tool supports concept normalization to UMLS CUIs or SNOMED CT codes, it does not provide sense disambiguation. In the future, we plan to evaluate the normalization performance and will address relation extraction tasks by annotating the corpus with relations.

### Usage

First, download the models from the Hugging Face hub. Then, import the AutoModelForTokenClassification class and load the NER model; for example, to annotate texts with the RoBERTa bsc-bio-ehr-es model for UMLS semantic groups, use: model = “roberta-es-clinical-trials-umls-7sgs-ner” (Figure C.18). Along with the graphical user interface (Fig. 1), the code can be executed via an UNIX-based terminal (Figure C.19) either for a single document or a batch of files included in a folder. A configuration file (Figure C.20) facilitates the specification of annotation options: e.g., use of the lexicon, temporal entities, negation or speculation, or output format of the annotations (JSON or BRAT ann). The code is available at: https://github.com/lcampillos/medspaner.

## Conclusions

We have introduced a comprehensive NLP tool designed to automate the processing of clinical trials in Spanish and demonstrated its efficacy in extracting medical information from clinical cases. The Medical Semantic Python-Assisted Named Entity Recognizer (MEDSPANER) is open source, supports both lexicon- and Transformer-based annotation of medical entities, and also normalizes entities to UMLS Concept Unique Identifiers or SNOMED CT codes. The tool integrates Transformer-based NER models that can be adapted to other sub-genres (e.g., clinical reports or medical journal articles). In addition, the models for negation or speculation and temporal entity recognition can be re-adapted for non-medical domains. We conducted an internal validation, with F1 scores up to 0.915 (strict match). We also performed an external validation in which eight medical professionals evaluated the system annotations on 100 new clinical trials texts (average F1 = 0.858) and 100 anonymized clinical cases (with an average F1 = 0.910). To the best of our knowledge, this is one of the few tools for comprehensive processing of Spanish medical texts, including the processing of medication information, experiencer and event temporality. The tool can be adapted to other languages for which similar text data and resources exist. We make the tool available online and distribute the code. Because new language models will be released, which are expected to surpass the results presented herein, we created a space at the Hugging Face Hub to share these and future models. We also release in a companion repository the annotated corpus for training the models.

## Supplementary Information

Supplementary Material 1.

## Figures and Tables

**Fig. 8:**
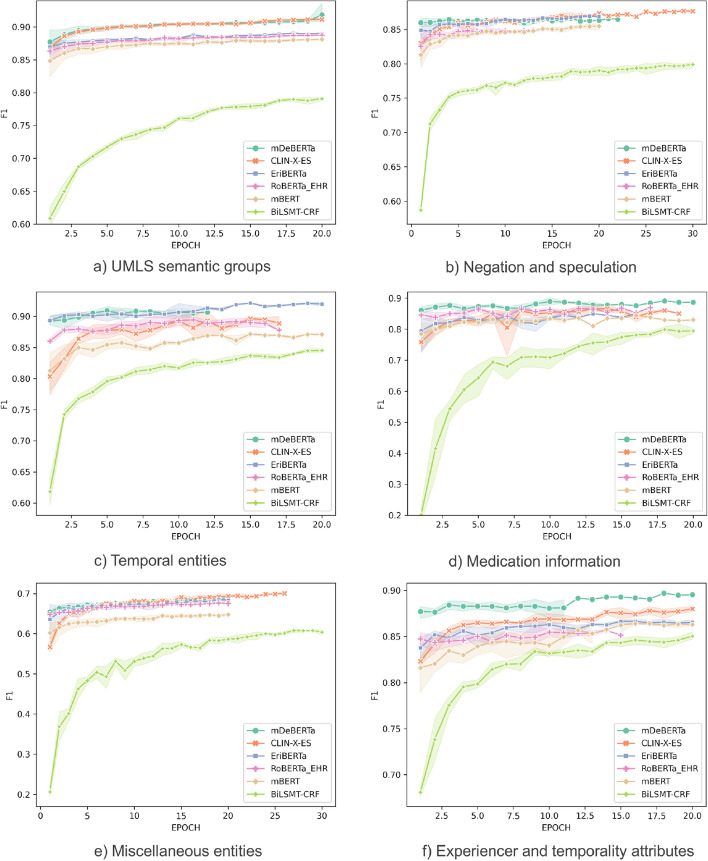
F1 measure (averaged over 5 runs) over training iterations (the shaded area represents standard deviation)

**Fig. 9:**
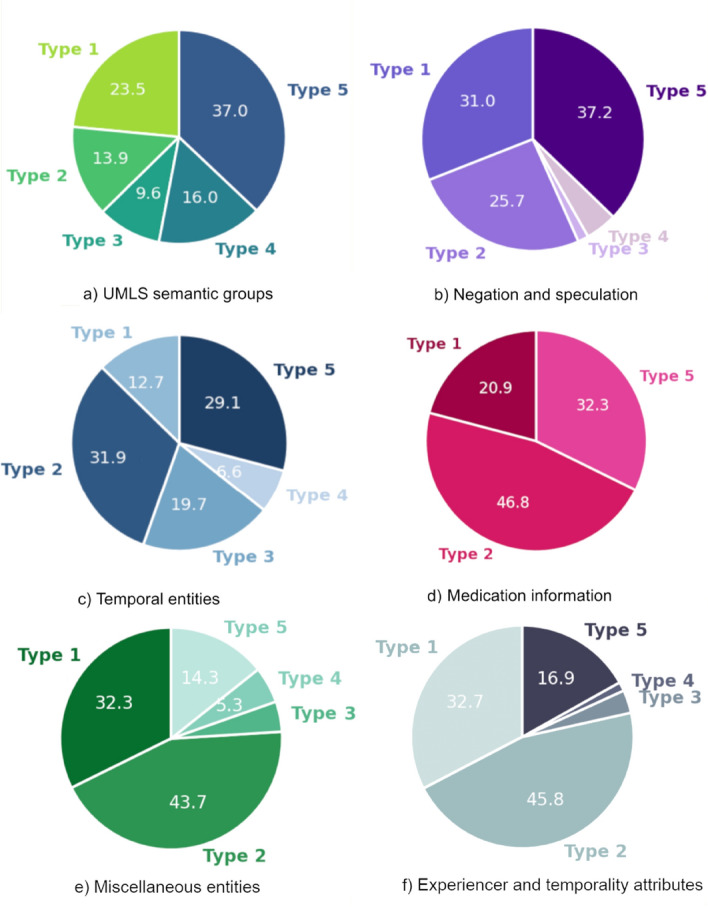
Errors (%) with RoBERTa EHR models classified according to [111]. Type 1: false positives; Type 2: false negatives; Type 3: same span, label mismatch; Type 4: overlapping span, wrong label; Type 5: overlapping span, right label

**Fig. 10:**
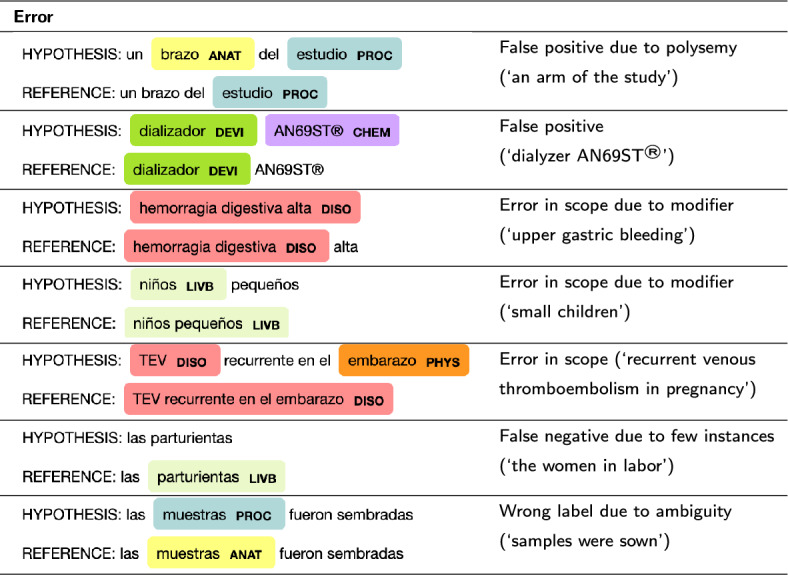
Samples of NER errors of UMLS semantic groups (translation in brackets)

**Fig. 11:**
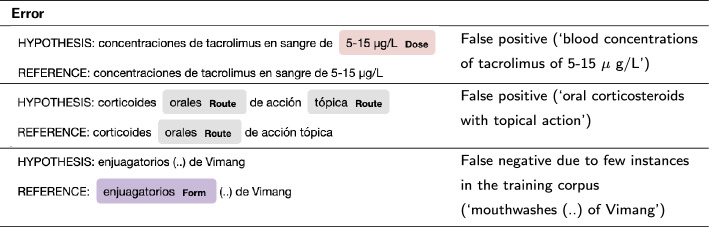
Samples of NER errors of medication information (translation in brackets)

**Fig. 12:**
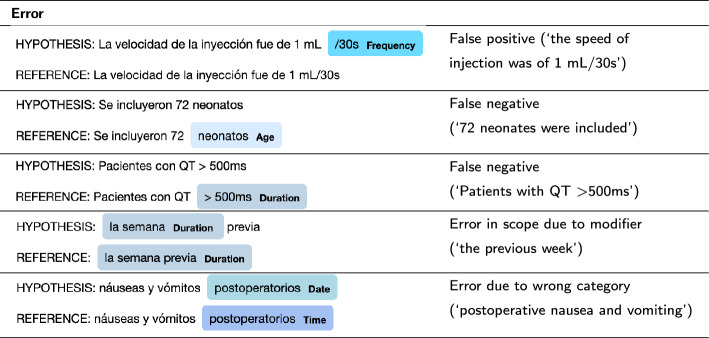
Samples of NER errors of temporal entities (translation in brackets)

**Fig. 13:**
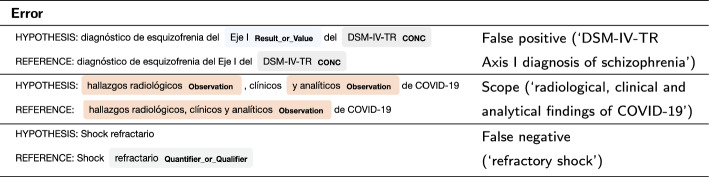
Samples of NER errors of miscellaneous entities (translation in brackets)

**Fig. 14:**
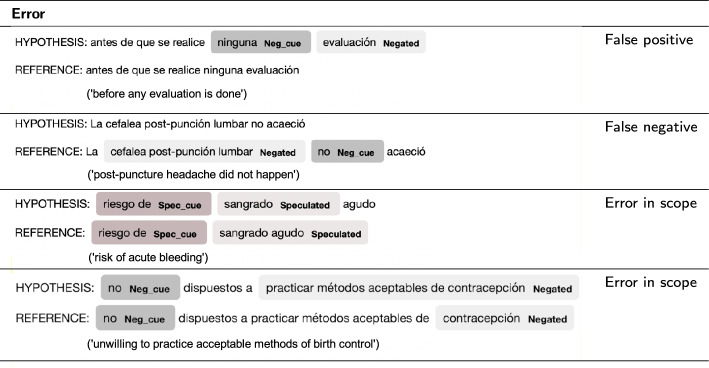
Samples of NER errors of negation and speculation (translation in brackets)

**Fig. 15:**
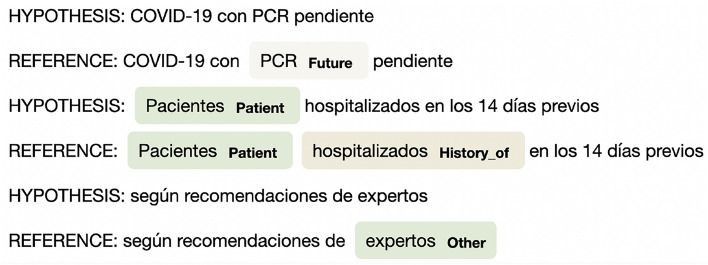
Samples of NER errors of event temporality and experiencer attributes (translation in brackets)

**Fig. 16:**
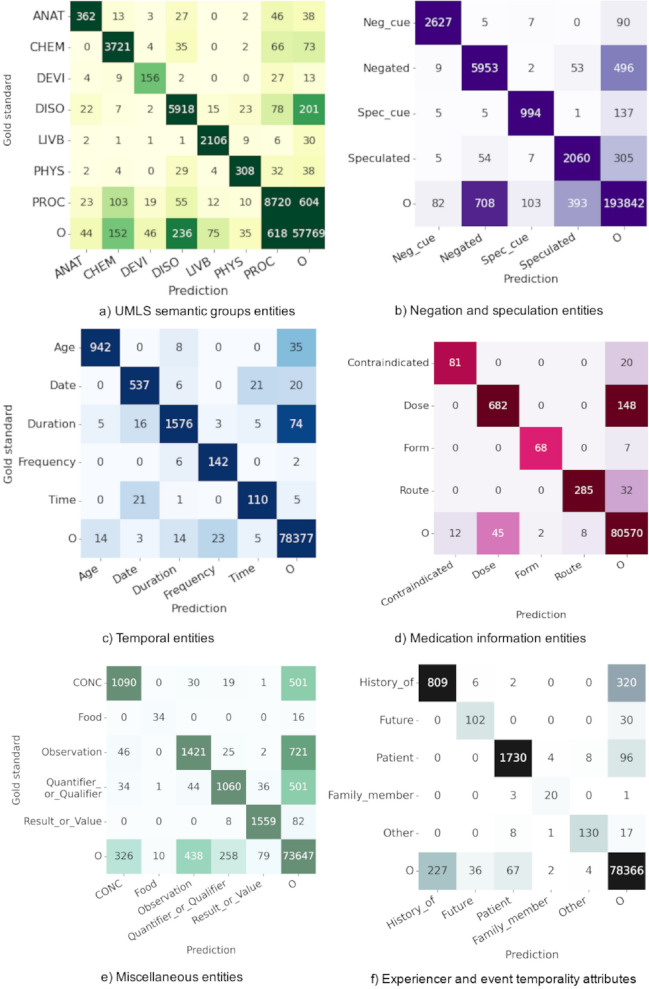
Confusion matrices of entity sub-classes, obtained with RoBERTa EHR Transformer-based model

**Fig. 17:**
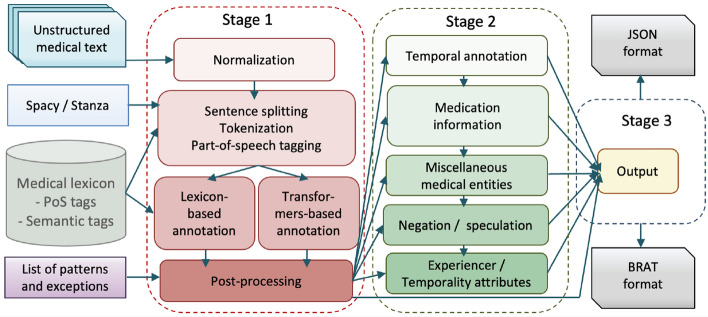
Processing pipeline

**Fig. 18:**
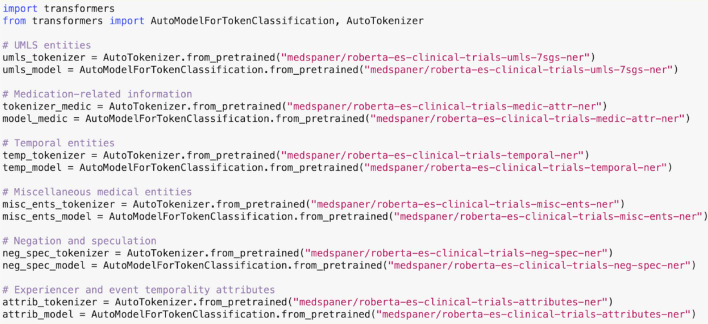
Sample code to import the Transformer-based models

**Fig. 19:**
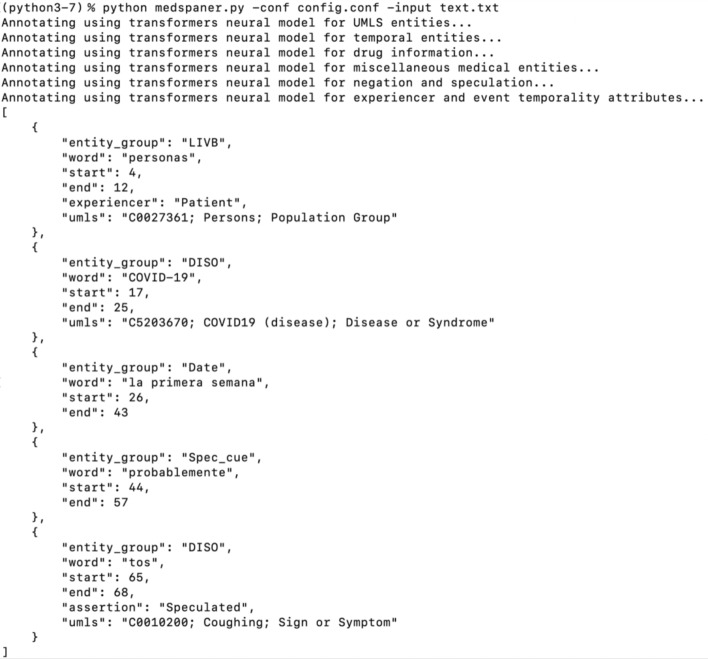
Annotation output from the UNIX-based terminal

**Fig. 20:**
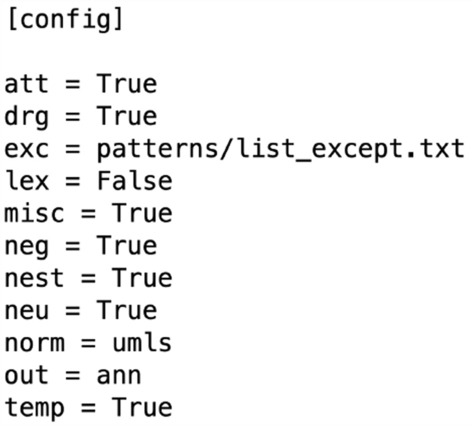
Configuration file with annotation options$^{*}$. $^{*}$att: annotate experiencer/temporality attributes; drg: annotate drug information; exc: use list of excluded words; lex: use lexicon; misc: annotate miscellaneous entities; neg: detect negation/speculation; nest: output nested entities; neu: UMLS neural models; norm: umls (if normalization to UMLS CUIs) or snomed (if normalization to SNOMED CT codes); out: output format (ann for BRAT, or json); temp: annotate temporal entities.

## Data Availability

All the resources supporting this article are available at the companion repository: https://github.com/lcampillos/medspaner. A demonstration system of the web interface is available at: https://claramed.csic.es/medspaner . The annotated corpus to train the models can be downloaded at: https://zenodo.org/records/13880599. The fine-tuned models are available at: https://huggingface.co/medspaner. .

## Appendix A. Training and annotation details

**Table 6:** Training epochs per model (average of five rounds and standard deviation)

Model	UMLS	Medication	Temporal	Negation/	Miscellaneous	Experiencer/
	entities	data	entities	Speculation	entities	Temporality
Bi-LSTM-CRF	95.60	68.40	68.20	80.60	78.80	71.20
	(± 5.37)	(± 7.47)	(± 7.29)	(± 15.07)	(± 9.44)	(± 7.73)
RoBERTa EHR	17.00	14.20	14.00	10.80	16.80	10.80
	(± 2.83)	(± 3.63)	(± 2.24)	(± 1.92)	(± 3.56)	(± 4.09)
EriBERTa	15.50	10.40	12.80	17.25	15.60	16.40
	(± 4.12)	(± 3.78)	(± 6.72)	(± 5.50)	(± 6.07)	(± 3.36)
CLIN-X-ES	17.00	14.60	13.60	21.80	19.40	16.40
	(± 2.83)	(± 3.65)	(± 4.22)	(± 5.36)	(± 6.07)	(± 4.83)
mBERT	14.75	11.40	14.00	16.00	18.20	11.60
	(± 6.18)	(± 5.22)	(± 5.61)	(± 6.16)	(± 2.49)	(± 2.07)
mDeBERTa vs 3	17.80	11.60	9.80	15.00	18.00	11.60
	(± 4.92)	(± 5.68)	(± 2.28)	(± 6.93)	(± 2.74)	(± 4.83)

**Table 7:** Annotation times (seconds per text) on 100 new trial texts and 100 clinical cases

Text	Average	Average	RoBERTa	EriBERTa	CLIN-X-ES	mDeBERTa
type	bytes/text	tokens/text	EHR			vs 3
EudraCT	2074.92	298.51	24”	24”	96”	153”
	(± 763.67)	(± 115.71)				
Clinical cases	1945.18	278.31	28”	29”	111”	129”
	(± 593.39)	(± 80.55)				

For each model architecture, we timed the annotation process in which six models were applied (for UMLS entities, medication information, temporal entities, negation/speculation, miscellaneous entities and experiencer/event temporality attributes)

### Appendix B. Detailed results and error analysis

**Table 8:** Results of the RoBERTa EHR model per entity type$^{*}$

UMLS	Precision	Recall	F1	Temporal	Precision	Recall	F1
entities				entities			
ANAT	0.728	0.686	0.706	Age	0.926	0.947	0.936
[ 308 ]	(± 0.030)	(± 0.030)	(± 0.025)	[ 372 ]	(± 0.013)	(± 0.009)	(± 0.010)
CHEM	0.917	0.923	0.920	Date	0.931	0.895	0.913
[ 2932 ]	(± 0.005)	(± 0.008)	(± 0.005)	[ 412 ]	(± 0.015)	(± 0.014)	(± 0.013)
DEVI	0.645	0.791	0.711	Duration	0.918	0.893	0.905
[ 134 ]	(± 0.018)	(± 0.047)	(± 0.027)	[ 629 ]	(± 0.014)	(± 0.019)	(± 0.010)
DISO	0.890	0.903	0.896	Frequency	0.780	0.885	0.829
[ 3065 ]	(± 0.008)	(± 0.003)	(± 0.003)	[ 73 ]	(± 0.043)	(± 0.008)	(± 0.024)
LIVB	0.949	0.959	0.954	Time	0.722	0.809	0.762
[ 1685 ]	(± 0.004)	(± 0.006)	(± 0.002)	[ 113 ]	(± 0.068)	(± 0.042)	(± 0.052)
PHYS	0.766	0.765	0.765	Miscellaneous	Precision	Recall	F1
[ 308 ]	(± 0.021)	(± 0.012)	(± 0.008)				
PROC	0.842	0.871	0.856	Concept	0.644	0.612	0.627
[ 4154 ]	(± 0.002)	(± 0.004)	(± 0.002)	[ 764 ]	(± 0.016)	(± 0.019)	(± 0.009)
Medication data	Precision	Recall	F1	Food/Drink	0.692	0.733	0.712
				[ 27 ]	(± 0.049)	(± 0.071)	(± 0.058)
Contraindicated.	0.818	0.816	0.812	Observation	0.626	0.617	0.621
[ 76 ]	(± 0.047)	(± 0.104)	(± 0.049)	[ 822 ]	(± 0.015)	(± 0.010)	(± 0.010)
Dose	0.824	0.843	0.833	Quant./Qual.	0.700	0.661	0.680
[ 314 ]	(± 0.039)	(± 0.020)	(± 0.021)	[ 1202 ]	(± 0.015)	(± 0.020)	(± 0.008)
Form	0.932	0.884	0.907	Result/Value	0.828	0.910	0.867
[ 74 ]	(± 0.030)	(± 0.020)	(± 0.019)	[ 394 ]	(± 0.013)	(± 0.005)	(± 0.007)
Route	0.916	0.899	0.907	Experiencer/temp. attr.	Precision	Recall	F1
[ 288 ]	(± 0.037)	(± 0.022)	(± 0.017)				
Negation/Speculation	Precision	Recall	F1	Family_member	0.721	0.920	0.808
				[ 20 ]	(± 0.048)	(± 0.027)	(± 0.034)
Neg_cue	0.955	0.958	0.957	Other	0.852	0.805	0.828
[ 2484 ]	(± 0.006)	(± 0.006)	(± 0.005)	[ 120 ]	(± 0.019)	(± 0.015)	(± 0.011)
Negated	0.829	0.837	0.833	Patient	0.949	0.921	0.935
[ 3160 ]	(± 0.005)	(± 0.014)	(± 0.008)	[ 1462 ]	(± 0.003)	(± 0.005)	(± 0.003)
Spec_cue	0.834	0.859	0.846	Future	0.640	0.620	0.629
[ 756 ]	(± 0.021)	(± 0.017)	(± 0.007)	[ 70 ]	(± 0.040)	(± 0.059)	(± 0.045)
Speculated	0.708	0.719	0.713	History_of	0.742	0.667	0.703
[ 1008 ]	(± 0.019)	(± 0.016)	(± 0.016)	[ 647 ]	(± 0.021)	(± 0.016)	(± 0.010)

$^{*}$ The number of instances of each type in the test set (support) appears in square brackets

(below each entity name in the first column); *temp. att.* stands for ‘temporality attributes’

### UMLS entities

Scope errors (type 5) were the most frequent. These especially affected adjectives that were not annotated in the gold standard, or vice versa (e.g., *hemorragia digestiva* *alta*, ‘upper gastric bleeding’; Fig. 10). They also occurred in proper names. Scope errors often appeared in compound entities, when the model predicted two separate entities (but one entity was marked the gold standard), or vice versa (e.g., ‘recurrent VTE in pregnancy’). The scope caused many ambiguities to the human annotators.

False positives (FPs, type 1 errors) abounded. These were related to polysemous entities (e.g., *brazo*, ‘arm’, was labeled as ANAT when it referred to each trial cohort). Some proper names and abbreviations were mislabeled; e.g., CHEM in *AN69ST*$^{\circledR }$, or *FCI* (‘informed consent document’) misinterpreted as DEVI. Most false negatives (FNs, type 2 errors) were owing to the lack of enough instances in the training corpus, namely anatomical entities, physiology terms and medical devices. Some infrequent abbreviations were not recognized (e.g., *GE*, ‘experimental group’, LIVB). Wrong labels (type 3 and 4 errors) were due to ambiguous entities: e.g., *muestras* can refer to the anatomic samples (ANAT) or to the sampling procedure (PROC).

### Medication information entities

Many errors were FPs (type 1). In particular, the model incorrectly labeled as Dose some number expressions and values of lab results (e.g., *blood concentrations of tacrolimus of 5-15 *$\mu$* g/L*). This mostly affected the eligibility criteria of trial announcements and was the main cause of the model’s decreased performance. Route FPs involved words expressing spatial concepts related to procedures. Semantically-related terms expressing the location of a drug agent also caused FPs (e.g., *oral corticosteroids with* *topical** action*).

However, the most frequent error were FNs (type 2). These mostly affected Form entities, since some dosage form terms were infrequent in the training data (e.g., *film, gel, mouthwashes*). Likewise, the model’s performance was not high on the Contraindicated class, given the scarce number of entities in the corpus. Errors in this category caused both FNs and FPs (e.g., the model also annotated Contraindicated in some diseases, whereas our annotation scheme only focused on medications or procedures). In most contexts, this was not a serious error. Scope errors (type 5) were very abundant and mostly affected quantifiers in Dose entities (e.g., *superior** a 10 mg*, ‘above 10 mg’). Mismatch errors (type 3 and 4) were sporadic (Fig. 11).

### Temporal entities

Most errors were FNs (type 2), namely in entities with low frequency in our training data (e.g., *pre-inclusion, QT > 500ms*; Fig. 12). Also, the model did not annotate Age in some LIVB entities expressing age groups (e.g., *neonate*). The second most common error affected the scope (type 5), namely when the model predicted a shorter span of the full entity (e.g., *la semana** previa*, ‘the previous week’).

Numerous errors were due to label mismatch (type 3), especially in Date and Time entities with *pre-* / *post-* affixes. Their meaning depends on the context: e.g., *postoperatorio* tends to be Date (as in *dolor postoperatorio*, ‘postoperative pain’), but it is Time when it refers to a period shorter than 24 h. Another confusion affected Date and Duration, which can be interpreted as a point in time, an interval or a temporal container (e.g. *the fifth week, over the past few years*). Those entity types were ambiguous for the human annotators (the IAA score of Time was low).

FPs (type 1) occurred in LIVB entities mislabeled as Age (e.g., *student*). Some numbers in protocol identifiers were mistaken as Date (e.g., *NOPHO-DBH AML* *2012** protocol*). Other errors were due to tokenization (e.g., *min-1* in Frequency) or inconsistencies in the gold standard.

### Miscellaneous entities

FNs (type 2) were the most recurrent errors, and tended to appear in Food_or_Drink, Observation or Quantifier_or_Qualifier entities (e.g. *refractory** shock*; Fig. 13). These entities are highly variable and some instances in the test set might not occur in the training data. FPs (type 1) are the second most prevalent errors, and affected Result_or_Value entities very often. Especially, when an expression contains numbers or typographical characters used in measurements: e.g. *DSM-IV-TR* *Axis I** diagnosis*. Finally, scope errors (type 5) were common in Observation entities. Often, the model did not label some parts of the entity, or tagged discontinuous expressions (while one entity was tagged in the gold standard): e.g. *radiological**, clinical **and analytical findings*. This can be related to the high variability of word forms and also to annotation inconsistencies.

### Negation and speculation

The main source of errors was related to scope (type 5). These occurred when the model did not label modifiers (or vice versa): e.g., *riesgo de sangrado* *agudo* (‘risk of acute bleeding’). At times, the model did not label the negation or speculation cue when postponed after the focus event (e.g., *post-puncture headache did* *not** happen*).

FPs (type 1) also affected the scope. In some contexts, the model annotated the full scope, but only the focus of negation or speculation was marked in the reference (e.g., *unwilling to practice acceptable methods of birth control*). Other FPs were due to the modifier *negative*, in cases such as *Serological tests negative against syphilis*. According to our criteria, we only labeled the negated entity (*syphilis*), since the focus of the negation is the test result (Fig. 14).

Another cause of FPs were negated entities in a wider negated context. In the gold standard, we did not annotate them since negating a negated entity makes a positive statement: e.g., in *patients without any hearing impairment except age-related pathology*, only *hearing impairment* was marked as negated in the gold standard. These were ambiguous to human annotators, but we tried to preserve the semantic coherence of the eligibility criteria.

Contexts where medical knowledge is needed caused errors. In *Forms of psoriasis other than chronic plaque-type (e.g., pustular, erythrodermic and guttate psoriasis)*, the model labeled the entities between brackets as negated. However, these are not hyponyms nor co-referent of *plaque psoriasis*, they should not be negated. Stylistic usage of negation was problematic; the model mislabeled contexts where no event was in fact negated: e.g., *antes de que se realice* *ninguna evaluación* (‘before any evaluation is done’).

FNs (type 2) were the third main cause of error. These were mainly due to the lack of training instances. Lexical negation caused many errors since the model did not recognize verbs or nouns negating an event. Compared to syntactic negation cues, lexical cues vary to a greater degree: e.g., *cesar* (‘to quit’), *descartar* (‘to rule out’), *retirada de* (‘withdrawal of’), *supresión* (‘elimination’), etc. Indeed, the human annotators hesitated about whether they should mark them or not. The larger number of errors in speculation cues can be explained because more variants exist in markers (compared to negation cues; e.g., *dudosamente*, ‘doubtfully’; *potencial*, ‘potential’), and in completive clauses with speculation verbs (e.g., *creemos que*, ‘we believe that’; *evaluar si*, ‘to evaluate whether’). This variance also affected verbs in conditional mood, which can also express uncertainty (e.g., *habría que descartar*, ‘should be discarded’).

### Event temporality and experiencer attributes

FNs (type 2) represented almost half of the errors, and these tend to occur in attributes with few instances in the test set: e.g. Future is missing in *COVID-19 with pending* *PCR*. FNs also occurred in entities with higher variability. For example, the Other person attribute might be a medical professional, a legal representative or a researcher: e.g. it is missing in *according to* *experts* *recommendations*. The History_of attribute was missing in contexts without the standard cue words, or if these appeared far from the focus word in the sentence.

FPs (type 1) were the second most common error type. They often involved temporality attributes, when the model predicted Future or History_of in a present condition. Lastly, the experiencer attribute was also mismatched in contexts where a medical professional was the participant in a clinical trial (Fig. 15).

### Appendix D: Comparison of system results with similar works

**Table 9:** Comparison of F-score in similar tasks (corpora in brackets); results from [22] referenced from [114]

	UMLS & medical entities	Negation/speculation
[4]	0.715	0.943 (negation), 0.859 (status)
[22]	0.70 [i2b2]	0.63 (negation)
[5]	0.795	0.984 (negation)
[124]	0.855 [i2b2]	0.905 [i2b2] (negation)
	0.874 [In-house corpus]	0.899 [In-house corpus] (negation)
[61]		0.955 (neg. cue), 0.89 (neg. scope)
[NUBEs]		0.829 (spec. cue), 0.746 (spec. scope)
[62]		0.85 (negation)
[95]	0.798 [Spanish CWLC]	
[63]		0.963 & 0.975 (cue, French)
		0.765 & 0.880 (scope, French)
[116]	0.658 [Chia] & 0.785 [FRD]	
[64]		0.95 (neg. cue), 0.92 (neg. scope)
[NUBEs]		0.84 (spec. cue), 0.80 (spec. scope)
[64]		0.90 (neg. cue), 0.84 (neg. scope)
[In-house cancer corpus]		0.81 (spec. cue), 0.74 (spec. scope)
[65]		0.786 (negation, best model)
[DIANN corpus]		0.765 (negation, authors’ model)
[125]	0.660 (disorders)	
[E3C corpus, Spanish]		
	Temporal entities	Medication information
[55]	0.726 [THYME] & 0.762 [i2b2]	
[120]	0.838 [i2b2]	
[82]	0.889 [Spanish BARR]	
[121]	0.824 (scope), 0.783 (class) [HourGlass]	
	0.851 (scope), 0.831 (class) [TempEval2]	
[83]	0.761 (strict), 0.912 (relaxed) [E3C]	
[126]	0.986 (Age), 0.934 (Frequency), 0.991 (Date)	0.970 (Dose)
[In-house cancer corpus]	0.898 (Duration), 0.933 (Implicit date)	
[122]		0.91–0.95
[66]		0.899
[67]		0.921 [n2c2 task 2]
	Miscellaneous entities	Experiencer/event temp.
[60]		0.760 (Historical);
[inhouse corpus]		1.000 (Experiencer)
[77]	0.800 (Finding) [SpRadIE corpus]	
[95]	0.622 (Finding)	0.918 (Family_member) [CWLC]
[117]	0.267 (Observation); 0.462 (Qualifier);	
	0.735 (Value) [Chia]	
[123]	0.747 (Observation); 0.618 (Modifier)	0.416 (Family_member) [LEAF]
	0.967 (Value) [Chia]	

## Abbreviations

BERT: Bidirectional Encoder Representations from Transformers
Bi-LSTM: BidirectionalLong-Short Term Memory
BIO: Begin, Inside, Out
CRF: Conditional Random Fields
CT: Clinical trials
CUI: Concept Unique Identifier
EBM: Evidence-Based Medicine
EHR: Electronic health record
EudraCT: European Clinical Trials Register
FN: False negative
FP: False positive
GPU: Graphical processing unit
H: Hidden state
I: Input
IAA: Inter-annotator agreement
K: Key
LLM: Large language model
M: Mean
NER: Named entity recognition
NLP: Natural language processing
OOV: Out-of-vocabulary
P: Precision
Q: Query
R: Recall
REEC: Repositorio Español de Estudios Clínicos
SciELO: Scientific Electronic Library Online
SD: Standard deviation
SG: Semantic group
SNOMED CT: Systematized Nomenclature of Medicine - Clinical Terms
TP: True positive
V: Value

## Footnotes

1 The Andalusian Society of Family and Medicine (SAMFyC) authorized us to use the cases they published. We also used clinical cases aimed at educational purposes from https://hipocampo.org/ and https://urgenciasbidasoa.wordpress.com/casos-clinicos-3/ [Accessed: 2024-23-02].
